# Supplementary material for: Molecular detection of Bartonella spp. DNA in dogs with hemangiosarcoma
Source: PLoS One. 2025 Apr 22;20(4):e0321806. doi: 10.1371/journal.pone.0321806 (PMC12013947; doi:10.1371/journal.pone.0321806)
Supplement: Attachment — Submitted filename: PONE-D-25-00356.pdf [file pone.0321806.s001.pdf]

PLOS ONE

PCR testing of fresh frozen tissues substantially improves molecular detection of Bartonella spp. DNA in dogs with hemangiosarcoma.

--Manuscript Draft--

**Manuscript Number:**	PONE-D-25-00356
**Article Type:**	Research Article
**Full Title:**	PCR testing of fresh frozen tissues substantially improves molecular detection of Bartonella spp. DNA in dogs with hemangiosarcoma.
**Short Title:**	PCR testing of fresh frozen tissues substantially improves molecular detection of Bartonella spp. DNA in dogs.
**Corresponding Author:**	Edward Bealmear Breitschwerdt, PhD<br>North Carolina State University DOCS: NC State University Department of Clinical Sciences<br>Fuquay Varina, NC UNITED STATES OF AMERICA
**Keywords:**	Bartonella; dog; hemangiosarcoma; preservation method; quantitative polymerase chain reaction; droplet digital polymerase chain reaction; fresh frozen; formalin; formalin-fixed paraffin-embedded
**Abstract:**	Introduction: The potential role of pathogens, particularly vector-transmitted infectious agents, as a cofactor or cause of neoplasia has not been intensively investigated. We previously reported a potential link between Bartonella spp. bacteremia and splenic hemangiosarcoma (HSA) in dogs living in the United States. The purpose of this study was to: 1/ further determine the prevalence of Bartonella spp. DNA in dogs with splenic HSA from throughout the United States; 2/ assess the impact of sample preservation method on Bartonella spp. DNA amplification using characterized tissue samples from dogs diagnosed with HSA.<br>Methods: In a blinded manner, we determined the presence of Bartonella spp. DNA in scrolls from biorepository formalin-fixed paraffin-embedded (FFPE) spleens from dogs living in three distant locations geographically transecting the United States. DNA extracted from non-lesional spleens (n = 249), nodular lymphoid hyperplasia spleens (n = 248), and splenic hemangiosarcoma (n = 330) were tested by quantitative polymerase chain reaction (qPCR), and droplet digital PCR (ddPCR). Subsequently, Bartonella PCR results from FFPE tissues and formalin-fixed tissues were compared using previously tested fresh frozen tissues from an additional 48 dogs with HSA.<br>Results: There was no significant difference in the proportion of Bartonella PCR positive FFPE tissues from dogs diagnosed with an alesional spleen, nodular lymphoid hyperplasia, and splenic hemangiosarcoma. Regardless of the histological diagnosis, the most common Bartonella species identified was B. henselae (32/38). Bartonella spp. DNA was detected in a significantly larger proportion of fresh frozen tissues compared to FFPE tissues, when tested by qPCR (22/48 versus 1/48; $p < 0.0001$) or ddPCR (19/48 versus 1/48; $p < 0.0001$). Using ddPCR, Bartonella DNA was more often amplified from formalin-fixed tissues compared to FFPE tissues (15/39 versus 1/39; $p < 0.0001$). The sensitivity of qPCR on FFPE samples and formalin-fixed samples, when comparing to fresh frozen samples as the reference standard, was 4.5% and 11.8%, respectively.<br>Conclusion: Due to decreased DNA amplification efficiency, FFPE scrolls should not be used for the detection of Bartonella infection in spleen samples from dogs with HSA. PCR testing of fresh frozen tissues substantially improves the detection of Bartonella spp. infection. If fresh frozen tissues are not available, formalin-fixed tissues should be tested with digital PCR to enhance Bartonella DNA detection.
**Order of Authors:**	Cynthia Robveille, DVM, Msc, Dipl. ECVP
	Ricardo G Maggi
	Erin Lashnits
	Taryn A Donovan
	Keith E Linder

Powered by Editorial Manager® and ProduXion Manager® from Aries Systems Corporation

<table>
<tr><td></td><td>Daniel P Regan</td></tr>
<tr><td></td><td>Kevin D Woolard</td></tr>
<tr><td></td><td>Edward B Breitschwerdt</td></tr>
<tr><td>Additional Information:</td><td></td></tr>
<tr><td>Question</td><td>Response</td></tr>
<tr><td>Financial Disclosure

Enter a financial disclosure statement that describes the sources of funding for the work included in this submission. Review the submission guidelines for detailed requirements. View published research articles from PLOS ONE for specific examples.

This statement is required for submission and will appear in the published article if the submission is accepted. Please make sure it is accurate.

Funded studies
Enter a statement with the following details:
• Initials of the authors who received each award
• Grant numbers awarded to each author
• The full name of each funder
• URL of each funder website
• Did the sponsors or funders play any role in the study design, data collection and analysis, decision to publish, or preparation of the manuscript?

Did you receive funding for this work?</td><td>Yes</td></tr>
<tr><td>Please add funding details.
as follow-up to "Financial Disclosure

Enter a financial disclosure statement that describes the sources of funding for the work included in this submission. Review the submission guidelines for detailed requirements. View published research articles from PLOS ONE for specific examples.

This statement is required for submission and will appear in the published article if</td><td>This research was supported by the American Kennel Club Canine Health Foundation (CHF Grant No. 02519, EBB and MB, http://www.akcchf.org/) and the State of North Carolina. The funders had no role in study design, data collection and analysis, decision to publish, or preparation of the manuscript.</td></tr>
</table>

Powered by Editorial Manager® and ProduXion Manager® from Aries Systems Corporation

the submission is accepted. Please make sure it is accurate.<br><br>**Funded studies**<br>Enter a statement with the following details:<br>• Initials of the authors who received each award<br>• Grant numbers awarded to each author<br>• The full name of each funder<br>• URL of each funder website<br>• Did the sponsors or funders play any role in the study design, data collection and analysis, decision to publish, or preparation of the manuscript?<br><br>Did you receive funding for this work?"	
Please select the country of your main research funder (please select carefully as in some cases this is used in fee calculation).<br>as follow-up to "**Financial Disclosure**<br><br>Enter a financial disclosure statement that describes the sources of funding for the work included in this submission. Review the submission guidelines for detailed requirements. View published research articles from *PLOS ONE* for specific examples.<br><br>This statement is required for submission and **will appear in the published article** if the submission is accepted. Please make sure it is accurate.<br><br>**Funded studies**<br>Enter a statement with the following details:<br>• Initials of the authors who received each award<br>• Grant numbers awarded to each author<br>• The full name of each funder<br>• URL of each funder website<br>• Did the sponsors or funders play any role in the study design, data collection and analysis, decision to publish, or preparation of the manuscript?	UNITED STATES - US

Did you receive funding for this work?"	
**Competing Interests**<br><br>Use the instructions below to enter a competing interest statement for this submission. On behalf of all authors, disclose any competing interests that could be perceived to bias this work—acknowledging all financial support and any other relevant financial or non-financial competing interests.<br><br>This statement is required for submission and **will appear in the published article** if the submission is accepted. Please make sure it is accurate and that any funding sources listed in your Funding Information later in the submission form are also declared in your Financial Disclosure statement.<br><br>View published research articles from *PLOS ONE* for specific examples.<br><br>**NO authors have competing interests**<br><br>Enter: *The authors have declared that no competing interests exist.*<br><br>**Authors with competing interests**<br><br>Enter competing interest details beginning with this statement:<br><br>*I have read the journal's policy and the authors of this manuscript have the following competing interests: [insert competing interests here]*<br><br>* typeset	In conjunction with Dr. S. Sontakke and North Carolina State University, Edward B. Breitschwerdt holds US Patent 7,115,385 Media and Methods for Cultivation of Microorganisms, which was issued on October 3rd, 2006. He is a co-founder, shareholder and Chief Scientific Officer for Galaxy Diagnostics, a company that provides advanced diagnostic testing for the detection of Bartonella spp. infections. Ricardo G. Maggi is a co-founder and the Chief Technical Officer for Galaxy Diagnostics Inc. All other authors declare no potential conflicts of interest with respect to the research, authorship, and/or publication of this article.
**Ethics Statement**<br><br>Enter an ethics statement for this submission. This statement is required if the study involved:<br><br>• Human participants<br>• Human specimens or tissue	Ethical approval was not required as this is a retrospective study using canine tissues either submitted for diagnostic pathology services to the Schwarzman Animal Medical Center, Colorado State University, and University of California, or from the biospecimen repository of the Canine Comparative Oncology and Genomics Consortium.

• Vertebrate animals or cephalopods<br>• Vertebrate embryos or tissues<br>• Field research<br><br>Write "N/A" if the submission does not require an ethics statement.<br><br>General guidance is provided below. Consult the submission guidelines for detailed instructions. **Make sure that all information entered here is included in the Methods section of the manuscript.**	

Powered by Editorial Manager® and ProduXion Manager® from Aries Systems Corporation

**Format for specific study types**<br><br>**Human Subject Research (involving human participants and/or tissue)**<br>• Give the name of the institutional review board or ethics committee that approved the study<br>• Include the approval number and/or a statement indicating approval of this research<br>• Indicate the form of consent obtained (written/oral) or the reason that consent was not obtained (e.g. the data were analyzed anonymously)<br><br>**Animal Research (involving vertebrate animals, embryos or tissues)**<br>• Provide the name of the Institutional Animal Care and Use Committee (IACUC) or other relevant ethics board that reviewed the study protocol, and indicate whether they approved this research or granted a formal waiver of ethical approval<br>• Include an approval number if one was obtained<br>• If the study involved *non-human primates*, add *additional details* about animal welfare and steps taken to ameliorate suffering<br>• If anesthesia, euthanasia, or any kind of animal sacrifice is part of the study, include briefly which substances and/or methods were applied<br><br>**Field Research**<br><br>Include the following details if this study involves the collection of plant, animal, or other materials from a natural setting:<br>• Field permit number<br>• Name of the institution or relevant body that granted permission	
**Data Availability**<br><br>Authors are required to make all data underlying the findings described fully available, without restriction, and from the time of publication. PLOS allows rare exceptions to address legal and ethical concerns. See the PLOS Data Policy and FAQ for detailed information.	Yes - all data are fully available without restriction

A Data Availability Statement describing where the data can be found is required at submission. Your answers to this question constitute the Data Availability Statement and **will be published in the article**, if accepted.<br><br>**Important:** Stating 'data available on request from the author' is not sufficient. If your data are only available upon request, select 'No' for the first question and explain your exceptional situation in the text box.<br><br>Do the authors confirm that all data underlying the findings described in their manuscript are fully available without restriction?	
**Describe where the data may be found in full sentences. If you are copying our sample text, replace any instances of XXX with the appropriate details.**<br><br>• If the data are **held or will be held in a public repository**, include URLs, accession numbers or DOIs. If this information will only be available after acceptance, indicate this by ticking the box below. For example: *All XXX files are available from the XXX database (accession number(s) XXX, XXX.).*<br>• If the data are all contained **within the manuscript and/or Supporting Information files**, enter the following: *All relevant data are within the manuscript and its Supporting Information files.*<br>• If neither of these applies but you are able to provide **details of access elsewhere**, with or without limitations, please do so. For example:<br><br>*Data cannot be shared publicly because of [XXX]. Data are available from the XXX Institutional Data Access / Ethics Committee (contact via XXX) for researchers who meet the criteria for access to confidential data.*<br><br>*The data underlying the results presented in the study are available from (include the name of the third party*	All relevant data are within the manuscript and its Supporting Information files.

and contact information or URL).<br>• This text is appropriate if the data are owned by a third party and authors do not have permission to share the data.<br><br>* typeset	
Additional data availability information:	

Manuscript

Click here to access/download;Manuscript;Manuscript 01062025.docx

PCR testing of fresh frozen tissues substantially improves molecular detection of *Bartonella* spp. DNA in dogs with hemangiosarcoma.

Cynthia Robveille[1], Ricardo G. Maggi[1],Erin Lashnits[2], Taryn A Donovan[3], Keith E Linder[4], Daniel P. Regan[5], Kevin D Woolard[6], Edward B. Breitschwerdt[1*]

[1] Department of Clinical Sciences, Intracellular Pathogens Research Laboratory, North Carolina State University - College of Veterinary Medicine, Raleigh, North Carolina, United States of America

[2] Department of Medical Sciences, University of Wisconsin-Madison - School of Veterinary Medicine, Madison, Wisconsin, United States of America

[3] Department of Anatomic Pathology, The Schwarzman Animal Medical Center, New York, New York, United States of America

[4] Department of Population Health and Pathobiology, North Carolina State University - College of Veterinary Medicine, Raleigh, North Carolina, United States of America

[5] Department. of Microbiology, Immunology and Pathology, Colorado State University - College of Veterinary Medicine and Biomedical Sciences, Fort Collins, Colorado, United States of America

[6] Departments of Pathology, Microbiology and Immunology, University of California-Davis - School of Veterinary Medicine, Davis, California, United States of America

* Corresponding author

E-mail: ebbreits@ncsu.edu (EBB)

Abstract

Introduction: The potential role of pathogens, particularly vector-transmitted infectious agents, as a cofactor or cause of neoplasia has not been intensively investigated. We previously reported a potential link between *Bartonella* spp. bacteremia and splenic hemangiosarcoma (HSA) in dogs living in the United States. The purpose of this study was to: 1/ further determine the prevalence of *Bartonella* spp. DNA in dogs with splenic HSA from throughout the United States; 2/ assess the impact of sample preservation method on *Bartonella* spp. DNA amplification using characterized tissue samples from dogs diagnosed with HSA.

Methods: In a blinded manner, we determined the presence of *Bartonella* spp. DNA in scrolls from biorepository formalin-fixed paraffin-embedded (FFPE) spleens from dogs living in three distant locations geographically transecting the United States. DNA extracted from non-lesional spleens (n = 249), nodular lymphoid hyperplasia spleens (n = 248), and splenic hemangiosarcoma (n = 330) were tested by quantitative polymerase chain reaction (qPCR), and droplet digital PCR (ddPCR). Subsequently, *Bartonella* PCR results from FFPE tissues and formalin-fixed tissues were compared using previously tested fresh frozen tissues from an additional 48 dogs with HSA.

Results: There was no significant difference in the proportion of *Bartonella* PCR positive FFPE tissues from dogs diagnosed with an alesional spleen, nodular lymphoid hyperplasia, and splenic hemangiosarcoma. Regardless of the histological diagnosis, the most common *Bartonella* species identified was *B. henselae* (32/38). *Bartonella* spp. DNA was detected in a significantly larger proportion of fresh frozen tissues compared to FFPE tissues, when tested by qPCR (22/48 versus 1/48; p <0.0001) or ddPCR (19/48 versus 1/48; p <0.0001). Using ddPCR, *Bartonella* DNA was more often amplified from formalin-fixed tissues compared to FFPE tissues (15/39 versus 1/39; p <0.0001). The sensitivity of qPCR on FFPE samples and formalin-fixed samples, when comparing to fresh frozen samples as the reference standard, was 4.5% and 11.8%, respectively.

Conclusion: Due to decreased DNA amplification efficiency, FFPE scrolls should not be used for the detection of *Bartonella* infection in spleen samples from dogs with HSA. PCR testing of fresh frozen tissues substantially improves the detection of *Bartonella* spp. infection. If fresh frozen tissues are not available,

formalin-fixed tissues should be tested with digital PCR to enhance *Bartonella* DNA detection.

Introduction

Bartonella spp. are facultative intracellular, potentially zoonotic, Gram-negative bacteria, that are most often transmitted by arthropod vectors or animal bites and scratches [1]. To date, more than 50 species have been described. In dogs, these pathogens have resulted in granulomatous inflammation [2], and non-neoplastic vasoproliferative diseases, such as peliosis hepatis [3] and cutaneous angiomatosis [4]. Angiogenetic factors, like vascular endothelial growth factor, play a role in the pathogenesis of these diseases [5]. In addition, *Bartonella* DNA has been amplified from canine cancers, specifically perivascular wall tumors [5] and hemangiosarcoma (HSA) [6]. The latter is a common neoplasm in dogs, likely originating from hematopoietic stem cells or endothelial progenitor cells [7]. It generally affects middle-aged and neutered dogs, with German Shepherd Dogs, Golden Retrievers and Labrador Retrievers being overrepresented [8]. Common primary sites include the spleen, right atrium/auricle, liver, and skin/subcutis [9]. The prognosis of visceral HSA is poor because of a high rate of early and widespread metastases *via* hematogenous spread and local seeding after tumor rupture [10].

In a previous study performed in North Carolina, *Bartonella* spp. DNA was amplified more commonly from dogs with HSA (13/50; 26%) compared to nodular lymphoid hyperplasia (NLH) (5/50; 10%) and histologically unremarkable spleen from specific-pathogen free dogs (0/8; 0%) [11]. In that study, *Bartonella* spp. DNA was detected using conventional and real-time quantitative PCR from approximately 25 mg of splenic tissue manually excised from formalin-fixed paraffin-embedded (FFPE) surgical biopsy samples. A more recent study showed 32% (24/74) of fresh frozen biopsy samples from splenic HSA in dogs were *Bartonella* spp. PCR positive [6]. The use of FFPE tissue blocks has become widely used in molecular studies, as it represents the most frequently available resource for retrospective studies requiring prior histologic examination. Nevertheless, formalin induces DNA fragmentation, cytosine deamination, and cross-linking, that may negatively affect molecular analyses [12].

The aim of this study was to assess the proportion of dogs with *Bartonella* spp. DNA in splenic HSA *versus* non-neoplastic splenic specimens from three institutional-based pathology repositories. Given the generated results, we also evaluated the impact of sample preservation method on *Bartonella* spp. detection in dogs with HSA, by comparing fresh frozen tissue, formalin-fixed tissue, and FFPE tissue from each dog. In addition to quantitative polymerase chain reaction (qPCR), we used droplet digital PCR (ddPCR). This technique is based on partitioning of a single sample into 20,000 water-in-oil droplets, then each droplet undergoes a PCR simultaneously. It allows for absolute quantitation of target DNA molecules using binomial Poisson statistics, without the requirement for a standard curve [13].

Materials and Methods

Sample Collection

Formalin-fixed paraffin-embedded canine splenic samples were retrieved from pathology archive storage facilities, using the database from three veterinary institutions across the United States: The Schwarzman Animal Medical Center in New York (samples processed between 2006 and 2018), Colorado State University (samples containing at least 70% of viable tissue and processed between 2009 and 2019), and University of California at Davis. Samples were composed of alesional spleen (*n* = 249), NLH (*n* = 248), and HSA (*n* = 330). A single block was available per dog, and an H&E section from each block was independently reviewed by a board-certified pathologist (KEL) to confirm the histologic diagnosis. Demographic information and travel history were not obtained.

Given the generated results, tissues from 48 dogs diagnosed with HSA banked by the biospecimen repository of the Canine Comparative Oncology and Genomics Consortium were selected based on previously published *Bartonella* molecular results using fresh tissues snap-frozen in liquid nitrogen [6, 14]. We randomly chose 26 dogs positive by qPCR and/or ddPCR, and 22 dogs negative by qPCR and ddPCR. Matched formalin-fixed tissue (stored in ethanol) and FFPE tissue were tested by qPCR and ddPCR. Formalin-fixed tissues were no longer available for 9 dogs.

DNA Extraction

FFPE tissues were deparaffinized using xylene and ethanol. DNA was extracted from three 50-µm sections of FFPE tissues and approximately 25 mg of formalin-fixed tissues using a Qiagen DNeasy® Blood and Tissue kit (Qiagen, Valencia, CA) following the manufacturer's instructions. To prevent cross-contamination, a new sterile scalpel blade (for formalin-fixed tissues) or a new microtome blade (for FFPE tissues) was used for each sample. Empty paraffin blocks were included and analyzed as controls for potential DNA carryover, as well as negative extraction controls. DNA yield and quality was assessed by spectrophotometry (Nanodrop, Wilmington, DE). The DNA was stored at -20 °C until needed.

Molecular analyses

Both qPCR and ddPCR was performed in a blinded manner using primers targeting the 16S-23S rRNA intergenic transcribed spacer (ITS) region of *Bartonella* genus in conjunction with a BsppITS500 FAM-Taq Man® probe (5′ FAM-GTTAGAGCGCGCGCTTGATAAG -IABkFQ 3′; IDT® DNA Technology, NC, USA). Oligonucleotides BsppITS325s (5′ CTTCAGATGATGATCCCAAGCCTTCTGGCG 3′) and BsppITS543as (5′ AATTGGTGGGCCTGGGAGGACTTG 3′) were used as forward and reverse primers, respectively, as previously described [15]. Negative and positive controls were prepared using 5 µl of DNA from previously characterized healthy or positive dogs (clinical cases), respectively.

For qPCR, amplification was performed in a 25-µl final volume reaction containing 12.5 µl of SsoAdvanced™ Universal Probes Supermix (Bio-Rad, Hercules, CA, USA), 0.2 µl of 100 µM of each forward primer, reverse primer, and TaqMan probe (IDT® DNA Technology, Coralville, IA), 6.9 µl of molecular grade water (Genesee Scientific, San Diego, CA, USA), and 5 µl of DNA from each sample tested. Quantitative PCR was performed in an CFX96® (Bio-Rad, Hercules, CA) under the following conditions: a single hot-start cycle at 95 °C for 3 min followed by 45 cycles of denaturing at 94 °C for 10 s, annealing at 68 °C for 10 s, and extension at 72 °C for 10 s. Amplification was completed by an additional cycle at 72 °C for 30 s. Positive amplification was assessed by analysis of detectable fluorescence signal vs

cycle threshold values. Sequencing of all positive samples was performed at GENEWIZ Inc. (Research Triangle Park, NC, USA). Bacterial species and strain were defined by comparing similarities with other sequences deposited in the GenBank database using BLAST version 2.0.

The 20 µL final ddPCR reaction consisted of 5 µL of ddPCR™ Multiplex Supermix (Bio-Rad, Hercules, CA, USA), 0.2 µL of 100 µM of each forward primer, reverse primer, and TaqMan probe (IDT® DNA Technology, Coralville, IA, USA), 8.1 µL of molecular–grade water, 5 µL of sample DNA, 0.3 µL of 300 mM Dithiothreitol (DTT), and 1 µL of HindIII DNA restriction enzyme. The ddPCR analysis was performed using a QX One Droplet Digital PCR (Bio-Rad, Hercules, CA, USA) system under the following amplification conditions: a single hot-start cycle at 95 °C for 10 min followed by 40 cycles of denaturing at 94 °C for 30 s and annealing at 62.9 °C for 1 min. A final extension at 98 °C was performed for 5 min. Fluorescent droplet detection and distribution readings were recorded in channel 1. Due to instrument design limitations, digital PCR droplets are not able to be sequenced.

For the second part of the study, digital PCR amplification of the dog housekeeping gene BRAF was performed as amplifiable DNA internal control using the oligonucleotides CaFeBRAF-15s (5′-TCAYGAAGACCTCACAGTAAAAATAGGT-3′) and CaFeBRAF-110as (5′-GATCCAGACAACTGTTCAAACTGATG-3′) as forward and reverse primers, respectively, and oligonucleotides CaFeBRAF-50 (5′-Cy5.5-GTCTAGCCACAGTGAAATCTCGATG-BHQ_3-3′) as fluorescent probe (IDT® DNA Technology, NC, USA) [16]. Partitions were recorded in the orange channel.

Study size

With a sample size of 240 samples per group (alesional spleen, NLH, and HSA), assuming based on previous literature [11] that >25% of HSA samples contained *Bartonella* spp. DNA, this study had 80% power to detect a statistically significant difference (alpha = 0.05) if the proportion of each control group containing *Bartonella* DNA was <15%.

When comparing sample preservation methods, assuming >50% of [reference group] contained *Bartonella* spp. DNA, this study had 80% power to detect a statistically significant difference (alpha = 0.05) if the proportion of [comparison group] containing *Bartonella* DNA was <20%.

Statistical methods

The proportion of FFPE spleen samples from each diagnosis group (alesional, NLH, HSA) that had *Bartonella* spp. DNA amplified by qPCR or ddPCR was calculated; those proportions were compared using a chi-squared test of independence. The proportions of samples from each preservation method (fresh frozen, formalin-fixed, and FFPE) that had *Bartonella* spp. DNA amplified by qPCR or ddPCR was calculated; those proportions were compared using a chi-squared test of independence (or Fisher's exact test for small sample sizes). To compare the proportions of samples that were *Bartonella* PCR positive across geographic locations, we used chi-squared tests for each diagnosis group (alesional spleen, NLH, HSA) for qPCR and ddPCR. A Bonferroni correction for multiple comparisons was used, so for these comparisons statistical significance was set at $p < 0.00833$. To determine agreement between tests on two different samples from the same dog, the kappa statistic was calculated [17]. Statistical significance was set at $p \leq 0.05$. All statistical analysis was performed in R v. 4.3.2 (R Core Team 2023).

Ethics statement

Ethical approval was not required as this is a retrospective study using canine tissues either submitted for diagnostic pathology services to the Schwarzman Animal Medical Center, Colorado State University, and University of California, or from the biospecimen repository of the Canine Comparative Oncology and Genomics Consortium.

Results

The proportion of FFPE tissues *Bartonella* spp. qPCR positive did not differ significantly between non-lesional spleen (13/249; 5.2%), NLH (14/248; 5.6%), and splenic HSA (11/330; 3.3%) ($p = 0.359754$) (Table 1). A similar trend with slightly higher proportion of positive samples was obtained with ddPCR

(10.4%, 9.7%, and 11.2%, respectively, Table 1) (p = 0.836694). For each microscopic diagnosis, there was a statistically significant difference in the proportion of samples *Bartonella* spp. PCR positive between geographical locations, based on the locations of the submitting veterinary institutions, with ddPCR (p = 0.0004 for alesional spleens, p = 0.0005 for NLH, and p = 0.0009 for HSA) but not qPCR (p = 0.0606 for alesional spleens, p = 0.3879 for NLH, and p = 0.1020 for HSA).

Table 1. Molecular results (qPCR, ddPCR) for the 827 FFPE tissues tested for *Bartonella* spp DNA.

**Diagnosis**	**Location**	**qPCR+**	**ddPCR+**	**qPCR+ and ddPCR+**	**qPCR+ or ddPCR+**
**normal spleen**	CSU (*n* = 90)	6	18	2	22 (24.4%)
	AMC (*n* = 66)	6	6	1	11 (16.7%)
	UC Davis (*n* = 93)	1	2	1	2 (2.1%)
	Total (*n* = 249)	**13 (5.2%)**	**26 (10.4%)**	**4 (1.6%)**	**35 (14.1%)**
**nodular lymphoid hyperplasia**	CSU (*n* = 76)	5	15	3	17 (22.4)
	AMC (*n* = 77)	6	1	1	6 (7.8%)
	UC Davis (*n* = 95)	3	8	1	10 (10.5%)
	Total (*n* = 248)	**14 (5.6%)**	**24 (9.7%)**	**5 (2.0%)**	**33 (13.3%)**
**hemangiosarcoma**	CSU (*n* = 111)	6	21	5	22 (19.8%)
	AMC (*n* = 88)	4	11	1	14 (15.9%)
	UC Davis (*n* = 131)	1	5	0	6 (4.6%)
	Total (*n* = 330)	**11 (3.3%)**	**37 (11.2%)**	**6 (1.8%)**	**42 (12.7%)**

CSU, Colorado State University; AMS, Animal Medical Center; UC, University of California

Regardless of the histological diagnosis, based upon DNA sequence analyses, the most common *Bartonella* species identified was *B. henselae* (32/38, including 1 co-infection with *B. quintana*-like in HSA). Other *Bartonella* species included: *B. quintana* in 1 alesional spleen and 1 NLH, *B. koehlerae* in 1 NLH, and *B. chomelii* in 1 HSA. *Bartonella* species could not be determined for 1 NLH and 1 HSA.

The molecular prevalence of *Bartonella* spp. was significantly higher in fresh frozen tissues compared to FFPE tissues, with both qPCR (22/48 versus 1/48; p <0.0001) and ddPCR (19/48 versus 1/48; p <0.0001), and in formalin-fixed tissues compared to FFPE tissues with ddPCR (15/39 versus 1/39; p <0.0001) (Table 2). There was qPCR amplification of *Bartonella* DNA in FFPE tissues spiked with *Bartonella henselae* (results not shown), supporting the absence of an inhibitor of DNA amplification. The sensitivity of qPCR on FFPE samples, when comparing to fresh frozen samples as the reference standard, was 4.5%. For dPCR, there was a larger number of partitions of the dog housekeeping gene (BRAFp) in the frozen samples compared to formalin-fixed tissues and FFPE tissues (results not shown).

Table 2. Molecular results (qPCR, ddPCR) for the 48 dogs diagnosed with HSA and selected on the basis of prior documentation of *Bartonella* spp PCR positivity or negativity. Residual formalin-fixed tissues were not available for 9 dogs.

	**Fresh frozen *vs* FFPE tissues (*n* = 48)**		**Fresh frozen *vs* FFPE *vs* Formalin-fixed tissues (*n* = 39)**		
qPCR+	22	1	17	0	5
ddPCR+	19	1	15	1	15
qPCR+ and ddPCR+	16	0	12	0	1
qPCR+ or ddPCR+	25	2	19	1	19

Bartonella spp. DNA was amplified by qPCR from a significantly higher proportion of fresh frozen tissues compared to formalin-fixed tissues (17/39 versus 5/39; p = 0.0025), but not when tested by ddPCR (15/39 for both; p = 1). The sensitivity of qPCR on formalin-fixed samples, when comparing to fresh frozen samples as the reference standard, was 11.8%.

For qPCR results, there was slight agreement between results on samples from the same dog when comparing FFPE and fresh frozen tissue samples (kappa = 0.05, 95% CI -0.04-0.14) or formalin-fixed and fresh frozen tissue samples (kappa = -0.02, 95% CI -0.25-0.21). The proportion of samples that did agree

(0.56 and 0.538, respectively) was not significantly higher than would be expected by chance alone (0.54, $p = 0.2719$; 0.548, $p = 0.8624$). For ddPCR results, there was slight agreement between results on samples from the same dog when comparing FFPE and fresh frozen tissue samples (kappa = -0.04, 95% CI -0.14-0.06). The proportion of samples that did agree (0.58) was not significantly higher than would be expected by chance alone (0.60, $p = 0.4134$). There was fair agreement between results on samples from the same dog when comparing formalin-fixed and fresh frozen tissue samples (kappa = 0.35, 95% CI 0.036-0.664). The proportion of samples that did agree (0.692) was significantly higher than would be expected by chance alone (0.527, $p = 0.0288$).

Discussion

Antemortem diagnosis of bartonellosis remains challenging due to the bacteria's fastidious and slow-growing nature, low and intermittent bacteremia, and the lack of sensitivity of serological techniques currently available. These limitations have proven to be substantial when assessing *Bartonella* spp. infection in dogs with histological confirmation of HSA [6, 14]. Also, due to current limitations with imaging modalities of *Bartonella* spp. with In Situ Hybridization and immunofluorescence, diagnosis of bartonellosis in surgical biopsy or postmortem tissue most often requires molecular testing [18-20]. Currently, despite known detrimental effects on PCR amplification sensitivity, many clinical, surgical and diagnostic workflows place ante- or post-mortem tissues solely into formalin for histologic evaluation, without consideration of the adverse effect in detection of microbial pathogens. As documented in this study, qPCR and ddPCR from FFPE tissues lowers *Bartonella* PCR sensitivity.

In this study, the proportion of samples with *Bartonella* positive qPCR from dogs diagnosed with splenic HSA or NLH from the three institutions was unexpectedly much lower compared to a previous study using FFPE tissues involving dogs with HSA from North Carolina [11]. As *Bartonella* infection in dogs appears to be ubiquitous across the United States [6], in retrospect, this discrepancy was likely related to differences in methodology between studies. While FFPE tissues were used in both studies, paraffin scrolls (150 µm thick) instead of histologically selected core punches (25 mg) were used in this study,

which resulted in a decreased tissue quantity per sample extraction. Thus, the quantity of extracted DNA (host and pathogen) potentially resulted in an underestimation of the actual proportion of *Bartonella* FFPE positive tissues. Because of DNA fragmentation in formalin-fixed paraffin-embedded tissues, the use of amplification products smaller than 120-150 bp is recommended to maximize the number of templates to be used for PCR [21, 22]. Targeting *Bartonella* ITS gene sequences, we identified *Bartonella henselae* as the most common *Bartonella* species, as previously reported, including one study using fresh frozen samples [6, 11]. As the generated amplicon for this species is around 140 bp, a minimal effect of DNA fragmentation for its detection is expected. Considering the rare flea infestations in Colorado, finding the highest proportion of samples *Bartonella* spp. ddPCR positive in the Colorado State University was surprising. Their samples had a short duration of fixation (≤ 24 hours) that could have positively impacted microbial detection.

The unexpectedly low amplification of *Bartonella* DNA from FFPE samples motivated our investigation of the impact of storage sample conditions in *Bartonella* spp. detection, using a previously characterized sample set [6]. Test results for matched FFPE and fresh tissues from 48 dogs, with a histological diagnosis of HSA, were compared for qPCR and dPCR sensitivity. Among the 22 *Bartonella* qPCR+ dogs using fresh frozen tissues, only 1 dog remained qPCR+ using scrolls of FFPE tissue. Consequently, PCR using DNA extracted from paraffin scrolls does not appear suitable for *Bartonella* molecular testing. The sensitivity limitation of scrolls has been described for the detection of another intracellular pathogen, *Mycobacterium tuberculosis* [23]. Using this dog sample set, the molecular prevalence of *Bartonella* spp. was significantly lower in formalin-fixed tissues compared to fresh frozen tissues with qPCR, but did not significantly differ with ddPCR. This result could be explained by the higher sensitivity, especially in case of low bacterial load [14, 24, 25] and higher inhibitor tolerance of the ddPCR compared to qPCR [26]. Indeed, the ratio between target DNA to PCR reagents is substantially higher in ddPCR compared to qPCR. ddPCR has already shown its utility in the accurate quantitation of bacteria [27, 28]. It is worth noting that the template DNA volume, as well as the primers and probe sequence and

concentrations, were identical for qPCR and ddPCR to avoid biases to the extent possible when comparing quantification of both assays. Obviously, by necessity, a different aliquot of extracted DNA had to be used for each comparative PCR reaction.

Interestingly, 6 of 15 dogs that were ddPCR+ in formalin-fixed tissues were previously ddPCR- in fresh frozen tissues. This finding was likely related to the low quantity and dispersed distribution of *Bartonella* within tissues, as well as testing only a single sample for each storage condition per animal. Using fresh frozen tissues, Lashnits et al. documented that *Bartonella* spp. DNA was ddPCR amplified from one of the two biopsy samples tested in 76% of bacteremic dogs with HSA, suggesting an increased diagnostic sensitivity of molecular testing when the latter is performed on multiple samples [14]. Similarly, sequential testing of human blood samples (obtaining 3 vs 1 sample) also increased the molecular detection of *Bartonella* spp. [29]. When attempting to confirm infection with a *Bartonella* sp., taking steps to optimize sensitivity of DNA detection should include testing multiple tissue locations and using duplicate from each tissue to accurately assess the prevalence.

FFPE DNA damage is influenced by many factors during processing and storage. Limitations in this study include unknown fixation time and duration between resection and formalin fixation, the variation in age of the paraffin blocks and storage parameters, all of which could have influenced the DNA quality and molecular results [30]. Different freezing methods were not assessed, as only samples snap-frozen in liquid nitrogen (subsequently stored at -80° C) were available. Also, despite testing matched samples, the quantity and distribution of *Bartonella* likely varied among tissues selected for DNA extraction, as reported previously in cats with endomyocarditis-left ventricular endocardial fibrosis and *Bartonella* spp. infections [31].

In conclusion, FFPE scrolls should not be used for the detection of *Bartonella* infection in spleen samples from dogs with HSA. PCR testing of fresh frozen tissues substantially improves the detection of *Bartonella* spp. infection. If fresh frozen tissues are not available, testing core formalin-fixed tissues with droplet digital PCR should improve sensitivity over testing FFPE tissues.

Acknowledgements

We thank Dr. Toni Richardson and Chance Liedig for their assistance in generating the molecular results in the Intracellular Pathogens Research Laboratory, North Carolina State University.

References

1. Breitschwerdt EB. Bartonellosis, One Health and all creatures great and small. Vet Dermatol. 2017;28(1):96-e21. doi: 10.1111/vde.12413. PubMed PMID: 28133871.

2. Pappalardo BL, Brown T, Gookin JL, Morrill CL, Breitschwerdt EB. Granulomatous disease associated with Bartonella infection in 2 dogs. Journal of veterinary internal medicine / American College of Veterinary Internal Medicine. 2000;14(1):37-42. Epub 2000/02/11. PubMed PMID: 10668815.

3. Kitchell BE, Fan TM, Kordick D, Breitschwerdt EB, Wollenberg G, Lichtensteiger CA. Peliosis hepatis in a dog infected with Bartonella henselae. Journal of the American Veterinary Medical Association. 2000;216(4):519-23, 7. Epub 2000/02/25. PubMed PMID: 10687006.

4. Yager JA, Best SJ, Maggi RG, Varanat M, Znajda N, Breitschwerdt EB. Bacillary angiomatosis in an immunosuppressed dog. Veterinary dermatology. 2010;21(4):420-8. Epub 2010/04/09. doi: 10.1111/j.1365-3164.2010.00879.x. PubMed PMID: 20374571.

5. Beerlage C, Varanat M, Linder K, Maggi RG, Cooley J, Kempf VA, et al. Bartonella vinsonii subsp. berkhoffii and Bartonella henselae as potential causes of proliferative vascular diseases in animals. Medical microbiology and immunology. 2012;201(3):319-26. Epub 2012/03/28. doi: 10.1007/s00430-012-0234-5. PubMed PMID: 22450733.

6. Lashnits E, Neupane P, Bradley JM, Richardson T, Thomas R, Linder KE, et al. Molecular prevalence of Bartonella, Babesia, and hemotropic Mycoplasma species in dogs with hemangiosarcoma from across the United States. PLoS One. 2020;15(1):e0227234. Epub 20200110. doi: 10.1371/journal.pone.0227234. PubMed PMID: 31923195; PubMed Central PMCID: PMCPMC6953799.

7. Kakiuchi-Kiyota S, Obert LA, Crowell DM, Xia S, Roy MD, Coskran TM, et al. Expression of Hematopoietic Stem and Endothelial Cell Markers in Canine Hemangiosarcoma. Toxicol Pathol. 2020;48(3):481-93. Epub 20200110. doi: 10.1177/0192623319897539. PubMed PMID: 31918642.

8. Robinson KL, Bryan ME, Atkinson ES, Keeler MR, Hahn AW, Bryan JN. Neutering is associated with developing hemangiosarcoma in dogs in the Veterinary Medical Database: An age and time-period matched case-control study (1964-2003). The Canadian veterinary journal La revue veterinaire canadienne. 2020;61(5):499-504. PubMed PMID: 32355348; PubMed Central PMCID: PMCPMC7155881.

9. Pearson GR, Head KW. Malignant haemangioendothelioma (angiosarcoma) in the dog. The Journal of small animal practice. 1976;17(11):737-45. doi: 10.1111/j.1748-5827.1976.tb06937.x. PubMed PMID: 1034853.

10. Oksanen A. Haemangiosarcoma in dogs. Journal of comparative pathology. 1978;88(4):585-95. doi: 10.1016/0021-9975(78)90012-9. PubMed PMID: 568633.

11. Varanat M, Maggi RG, Linder KE, Breitschwerdt EB. Molecular prevalence of Bartonella, Babesia, and hemotropic Mycoplasma sp. in dogs with splenic disease. J Vet Intern Med. 2011;25(6):1284-91. Epub 20111012. doi: 10.1111/j.1939-1676.2011.00811.x. PubMed PMID: 22092618.

12. Do H, Dobrovic A. Sequence artifacts in DNA from formalin-fixed tissues: causes and strategies for minimization. Clinical chemistry. 2015;61(1):64-71. Epub 20141124. doi: 10.1373/clinchem.2014.223040. PubMed PMID: 25421801.

13. Hindson CM, Chevillet JR, Briggs HA, Gallichotte EN, Ruf IK, Hindson BJ, et al. Absolute quantification by droplet digital PCR versus analog real-time PCR. Nat Methods. 2013;10(10):1003-5. Epub 20130901. doi: 10.1038/nmeth.2633. PubMed PMID: 23995387; PubMed Central PMCID: PMCPMC4118677.

14. Lashnits E, Neupane P, Bradley JM, Richardson T, Maggi RG, Breitschwerdt EB. Comparison of Serological and Molecular Assays for Bartonella Species in Dogs with Hemangiosarcoma. Pathogens. 2021;10(7). Epub 20210623. doi: 10.3390/pathogens10070794. PubMed PMID: 34201572; PubMed Central PMCID: PMCPMC8308881.

15. Maggi RG, Richardson T, Breitschwerdt EB, Miller JC. Development and validation of a droplet digital PCR assay for the detection and quantification of Bartonella species within human clinical samples. Journal of microbiological methods. 2020;176:106022. Epub 2020/08/17. doi: 10.1016/j.mimet.2020.106022. PubMed PMID: 32795640.

16. Maggi R, Breitschwerdt EB, Qurollo B, Miller JC. Development of a Multiplex Droplet Digital PCR Assay for the Detection of Babesia, Bartonella, and Borrelia Species. Pathogens. 2021;10(11). Epub 20211111. doi: 10.3390/pathogens10111462. PubMed PMID: 34832618; PubMed Central PMCID: PMCPMC8620149.

17. McHugh ML. Interrater reliability: the kappa statistic. Biochem Med (Zagreb). 2012;22(3):276-82. PubMed PMID: 23092060; PubMed Central PMCID: PMCPMC3900052.

18. Saunders GK, Monroe WE. Systemic granulomatous disease and sialometaplasia in a dog with Bartonella infection. Vet Pathol. 2006;43(3):391-2. doi: 10.1354/vp.43-3-391. PubMed PMID: 16672593.

19. Caponetti GC, Pantanowitz L, Marconi S, Havens JM, Lamps LW, Otis CN. Evaluation of immunohistochemistry in identifying Bartonella henselae in cat-scratch disease. Am J Clin Pathol. 2009;131(2):250-6. doi: 10.1309/AJCPMNULMO9GPLYU. PubMed PMID: 19141385.

20. Edouard S, Nabet C, Lepidi H, Fournier PE, Raoult D. Bartonella, a common cause of endocarditis: a report on 106 cases and review. J Clin Microbiol. 2015;53(3):824-9. Epub 20141224. doi: 10.1128/JCM.02827-14. PubMed PMID: 25540398; PubMed Central PMCID: PMCPMC4390654.

21. Funabashi KS, Barcelos D, Visona I, e Silva MS, e Sousa ML, de Franco MF, et al. DNA extraction and molecular analysis of non-tumoral liver, spleen, and brain from autopsy samples:

the effect of formalin fixation and paraffin embedding. Pathol Res Pract. 2012;208(10):584-91. Epub 20120821. doi: 10.1016/j.prp.2012.07.001. PubMed PMID: 22920941.

22. Hockney R, Orr CH, Waring GJ, Christiaens I, Taylor G, Cummings SP, et al. Formalin-Fixed Paraffin-Embedded (FFPE) samples are not a beneficial replacement for frozen tissues in fetal membrane microbiota research. PloS one. 2022;17(3):e0265441. Epub 20220317. doi: 10.1371/journal.pone.0265441. PubMed PMID: 35298530; PubMed Central PMCID: PMCPMC8929612.

23. Heinmoller E, Renke B, Beyser K, Dietmaier W, Langner C, Ruschoff J. Piffalls in diagnostic molecular pathology--significance of sampling error. Virchows Arch. 2001;439(4):504-11. doi: 10.1007/s004280100450. PubMed PMID: 11710637.

24. Cao Z, Wu W, Wei H, Gao C, Zhang L, Wu C, et al. Using droplet digital PCR in the detection of Mycobacterium tuberculosis DNA in FFPE samples. International journal of infectious diseases : IJID : official publication of the International Society for Infectious Diseases. 2020;99:77-83. Epub 20200730. doi: 10.1016/j.ijid.2020.07.045. PubMed PMID: 32738487.

25. Doi H, Takahara T, Minamoto T, Matsuhashi S, Uchii K, Yamanaka H. Droplet digital polymerase chain reaction (PCR) outperforms real-time PCR in the detection of environmental DNA from an invasive fish species. Environ Sci Technol. 2015;49(9):5601-8. Epub 20150420. doi: 10.1021/acs.est.5b00253. PubMed PMID: 25850372.

26. Yang R, Paparini A, Monis P, Ryan U. Comparison of next-generation droplet digital PCR (ddPCR) with quantitative PCR (qPCR) for enumeration of Cryptosporidium oocysts in faecal samples. Int J Parasitol. 2014;44(14):1105-13. Epub 20140916. doi: 10.1016/j.ijpara.2014.08.004. PubMed PMID: 25229177.

27. Verhaegen B, De Reu K, De Zutter L, Verstraete K, Heyndrickx M, Van Coillie E. Comparison of Droplet Digital PCR and qPCR for the Quantification of Shiga Toxin-Producing Escherichia coli in Bovine Feces. Toxins (Basel). 2016;8(5). Epub 20160518. doi: 10.3390/toxins8050157. PubMed PMID: 27213452; PubMed Central PMCID: PMCPMC4885071.

28. Witte AK, Fister S, Mester P, Schoder D, Rossmanith P. Evaluation of the performance of quantitative detection of the Listeria monocytogenes prfA locus with droplet digital PCR. Anal Bioanal Chem. 2016;408(27):7583-93. Epub 20160824. doi: 10.1007/s00216-016-9861-9. PubMed PMID: 27558101; PubMed Central PMCID: PMCPMC5061835.

29. Pultorak EL, Maggi RG, Mascarelli PE, Breitschwerdt EB. Serial testing from a 3-day collection period by use of the Bartonella Alphaproteobacteria growth medium platform may enhance the sensitivity of Bartonella species detection in bacteremic human patients. Journal of clinical microbiology. 2013;51(6):1673-7. Epub 2013/03/15. doi: 10.1128/JCM.00123-13. PubMed PMID: 23486720; PubMed Central PMCID: PMC3716093.

30. Guyard A, Boyez A, Pujals A, Robe C, Tran Van Nhieu J, Allory Y, et al. DNA degrades during storage in formalin-fixed and paraffin-embedded tissue blocks. Virchows Arch. 2017;471(4):491-500. Epub 20170815. doi: 10.1007/s00428-017-2213-0. PubMed PMID: 28812131.

31. Donovan TA, Balakrishnan N, Carvalho Barbosa I, McCoy T, Breitschwerdt EB, Fox PR. Bartonella spp. as a Possible Cause or Cofactor of Feline Endomyocarditis-Left Ventricular Endocardial Fibrosis Complex. J Comp Pathol. 2018;162:29-42. Epub 20180704. doi: 10.1016/j.jcpa.2018.05.002. PubMed PMID: 30060840.

Fundings

This research was supported by the American Kennel Club Canine Health Foundation (CHF Grant No. 02519, EBB and MB, http://www.akcchf.org/) and the State of North Carolina. The funders had no role in study design, data collection and analysis, decision to publish, or preparation of the manuscript.

requested document 1 - dual publication
Click here to access/download;Manuscript;Lashnits et al 2020.pdf

RESEARCH ARTICLE

Molecular prevalence of *Bartonella*, *Babesia*, and hemotropic *Mycoplasma* species in dogs with hemangiosarcoma from across the United States

Erin Lashnits[1], **Pradeep Neupane**[1], **Julie M. Bradley**[1], **Toni Richardson**[1], **Rachael Thomas**[2], **Keith E. Linder**[3], **Matthew Breen**[2], **Ricardo G. Maggi**[1,4], **Edward B. Breitschwerdt**[1,4]*

1 Intracellular Pathogens Research Laboratory, Comparative Medicine Institute, College of Veterinary Medicine, North Carolina State University, Raleigh, North Carolina, United States of America, **2** Department of Molecular Biomedical Sciences, Comparative Genomics, College of Veterinary Medicine, North Carolina State University, Raleigh, North Carolina, United States of America, **3** Department of Population Health and Pathobiology, College of Veterinary Medicine, North Carolina State University, Raleigh, North Carolina, United States of America, **4** Department of Clinical Sciences, College of Veterinary Medicine, North Carolina State University, Raleigh, North Carolina, United States of America

* ebbreits@ncsu.edu

Check for updates

OPEN ACCESS

Citation: Lashnits E, Neupane P, Bradley JM, Richardson T, Thomas R, Linder KE, et al. (2020) Molecular prevalence of *Bartonella*, *Babesia*, and hemotropic *Mycoplasma* species in dogs with hemangiosarcoma from across the United States. PLoS ONE 15(1): e0227234. https://doi.org/10.1371/journal.pone.0227234

Editor: Douglas H. Thamm, Colorado State University, UNITED STATES

Received: October 3, 2019

Accepted: December 13, 2019

Published: January 10, 2020

Copyright: © 2020 Lashnits et al. This is an open access article distributed under the terms of the Creative Commons Attribution License, which permits unrestricted use, distribution, and reproduction in any medium, provided the original author and source are credited.

Data Availability Statement: All relevant data are available from Dryad at https://doi.org/10.5061/dryad.ttdz08ktd.

Funding: This research was supported by the American Kennel Club Canine Health Foundation (CHF Grant No. 2550, EBB and MB, http://www.akcchf.org/) and the State of North Carolina. EL's research is supported in part by the Comparative Medicine and Translational Research Program of the National Institutes of Health under award

Abstract

Hemangiosarcoma (HSA), a locally invasive and highly metastatic endothelial cell neoplasm, accounts for two-thirds of all cardiac and splenic neoplasms in dogs. *Bartonella* spp. infection has been reported in association with neoplastic and non-neoplastic vasoproliferative lesions in animals and humans. The objective of this study was to determine the prevalence of *Bartonella* spp. in conjunction with two other hemotropic pathogens, *Babesia* spp. and hemotropic *Mycoplasma* spp., in tissues and blood samples from 110 dogs with histopathologically diagnosed HSA from throughout the United States. This was a retrospective, observational study using clinical specimens from 110 dogs with HSA banked by the biospecimen repository of the Canine Comparative Oncology and Genomics Consortium. Samples provided for this study from each dog included: fresh frozen HSA tumor tissue (available from n = 100 of the 110 dogs), fresh frozen non-tumor tissue (n = 104), and whole blood and serum samples (n = 108 and 107 respectively). Blood and tissues were tested by qPCR for *Bartonella*, hemotropic *Mycoplasma*, and *Babesia* spp. DNA; serum was tested for *Bartonella* spp. antibodies. *Bartonella* spp. DNA was amplified and sequenced from 73% of dogs with HSA (80/110). In contrast, hemotropic *Mycoplasma* spp. DNA was amplified from a significantly smaller proportion (5%, $p<0.0001$) and *Babesia* spp. DNA was not amplified from any dog. Of the 100 HSA tumor samples submitted, 34% were *Bartonella* PCR positive (32% of splenic tumors, 57% of cardiac tumors, and 17% of other tumor locations). Of 104 non-tumor tissues, 63% were *Bartonella* PCR positive (56% of spleen samples, 93% of cardiac samples, and 63% of skin/subcutaneous samples). Of dogs with *Bartonella* positive HSA tumor, 76% were also positive in non-tumor tissue. *Bartonella* spp. DNA was not PCR amplified from whole blood. This study documented a high prevalence of *Bartonella* spp. DNA in dogs with HSA from geographically diverse regions of the United States. While 73%

number T32OD011130. The funders had no role in study design, data collection and analysis, decision to publish, or preparation of the manuscript.

Competing interests: In conjunction with Dr. Sushama Sontakke and North Carolina State University, Edward B. Breitschwerdt, DVM holds U. S. Patent No. 7,115,385: "Media and Methods for Cultivation of Microorganisms", which was issued October 3, 2006. He is a co-founder, shareholder and Chief Scientific Officer for Galaxy Diagnostics, a company that provides advanced diagnostic testing for the detection of Bartonella species infections. This does not alter our adherence to PLOS ONE policies on sharing data and materials. All other authors declare no potential conflicts of interest.

of all tissue samples from these dogs were PCR positive for *Bartonella* DNA, none of the blood samples were, indicating that whole blood samples do not reflect tissue presence of this pathogen. Future studies are needed to further investigate the role of *Bartonella* spp. in the development of HSA.

Introduction

There are clear precedents for the involvement of bacterial infection in neoplastic development. Within the past 25 years, a considerable volume of research has been conducted on the oncogenic properties of infectious agents such as bacteria, mycoplasma, protozoa, and viruses. [1,2] Currently, infectious agents are accepted as a cause or co-factor in anywhere from 5–50% of human cancers worldwide, depending on the geographic region and its development status. [1–3] The involvement of infectious agents in the pathogenesis of some human cancers is therefore well established. The majority of infectious agents implicated in oncogenesis are viruses, such as Epstein Barr virus, human papillomaviruses, and Kaposi's sarcoma-associated herpesvirus.[1] These viruses have direct oncogenic properties through integration of viral genomes into host cells, or by secretion of gene products into healthy cells to create tumor cells. The extent to which other infectious agents, such as bacteria, lack the inherent oncogenic properties of their viral counterparts remains unclear. Bacteria most often promote cancer development indirectly through persistent replication, inflammation and chronic tissue damage.[4,5] *Helicobacter pylori*, for example, colonizes and replicates within the gastric mucosa, resulting in a chronic pro-inflammatory response that promotes cancer risk and oncogenesis of gastric cancer by altering epithelial cell proliferation and apoptosis.[6] With the difficulty of assessing causality, particularly for certain rare cancer types, there may be roles for other pathogenic bacteria in a range of different cancers that have not yet been discovered.[7]

Hemangiosarcoma (HSA) is a highly aggressive endothelial cell cancer that is associated with local invasiveness and a high metastatic potential in dogs (Fig 1).[8,9] The most common neoplasm of the spleen,[10] this cancer is found in up to 2% of all dogs with tissues submitted for autopsy.[11] While HSA may affect any dog breed, several of the most popular family owned pure breeds are highly predisposed, including the golden retriever, Labrador retriever and German Shepherd Dog.[12–14] Splenic HSA is among the most common canine cancers encountered in clinical practice, accounting for approximately two thirds of all splenic tumors (neoplasms of the spleen, benign or malignant, comprise approximately half of all splenic pathology in dogs).[9] Cardiac HSA is less common than splenic HSA (most studies reporting less than 1% of all canine neoplasms), but remains the most common cardiac tumor of dogs. [15,16] Other primary tumor locations such as the liver and skin are reported, but rare.

Splenic HSA is often difficult to diagnose without resorting to splenectomy, a major invasive abdominal surgery.[8,17] Moreover, as an indolent disease, malignant HSA masses that develop within the abdominal cavity often remain undetected until reaching an advanced stage, at which time there is a high risk of spontaneous rupture potentially leading to untreatable and ultimately fatal internal hemorrhage.[18] Because of this risk, as well as the lack of broadly effective chemotherapeutics, the prognosis is poor. The median survival after diagnosis ranges from less than 3 weeks with splenectomy surgery alone to six months with surgery plus cancer chemotherapy.[19,20] As a result there is a critical need for improved diagnostic modalities for earlier detection of HSA, as well as new treatments and preventative strategies to improve outcomes for this common tumor of family pets.

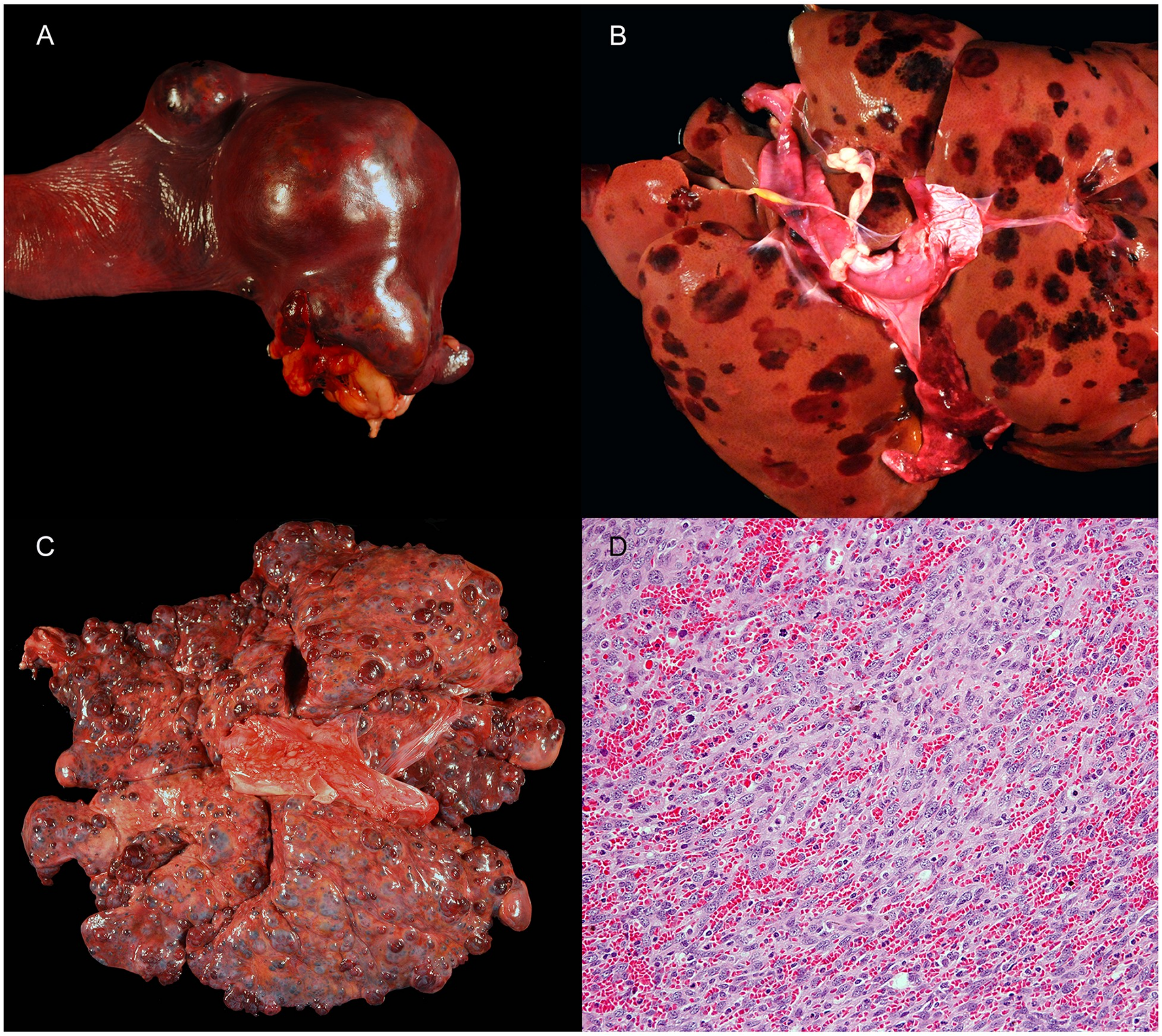

Fig 1. Images of hemangiosarcoma (HSA) tumors from dogs. A) Primary HSA mass in a spleen. B) Metastatic HSA in a liver with multifocal masses in all lobes. C) Metastatic hemangiosarcoma in the lungs with multifocal masses in all lobes. D) Photomicrograph of a splenic HSA from a dog in the current study that was PCR positive for *B. henselae*. 20X magnification. Hematoxylin and eosin. Credits: Talley A (image A), Sommer S (image B), Rasche B (image C) and Barnes J (image D).

https://doi.org/10.1371/journal.pone.0227234.g001

Despite substantial research, the etiology and pathogenesis of canine HSA remains unclear. As a malignant tumor of vascular endothelial cells, factors that have been hypothesized to contribute to the pathogenesis of HSA include chronic inflammation, macrophage activation, hypoxia, and angiogenesis.[21] *In vitro*, HSA cells produce growth factors promoting angiogenesis, including vascular endothelial growth factor-A (VEGF-A), platelet-derived growth factor-β (PDGF-β), and basic fibroblast growth factor (bFGF), and genes involved in inflammation, angiogenesis, and cellular adhesion and invasion can distinguish HSA cells from non-malignant endothelial cells.[9,21]

Because of established links between chronic intracellular infections, inflammation, and angiogenesis, efforts are being made to determine if chronic intravascular infection with bacteria or protozoa could contribute to HSA development in dogs. One previous study from our

laboratory examined the molecular prevalence of blood (erythrocyte)-borne pathogens in a small cohort of dogs from the southeastern United States with and without splenic pathology. [22] We found that *Bartonella* spp. were significantly more common than *Babesia* spp. or hemotropic *Mycoplasma* spp. in formalin-fixed, paraffin embedded biopsy samples from splenic HSA: 26% of dogs were positive for *Bartonella* spp. compared to 2% for *Babesia* spp. ($p < 0.001$) and 6% for hemotropic *Mycoplasma* spp. ($p = 0.006$). Moreover, *Bartonella* spp. were found more often in splenic HSA biopsy samples compared to samples from a non-neoplastic inflammatory disorder of the spleen (lymphoid nodular hyperplasia, LNH) and histologically normal splenic tissue from specific-pathogen-free dogs.[22] We have subsequently documented that *Bartonella* spp. DNA can be amplified from angioproliferative lesions in cats, cows, dogs and horses.[23] In addition, it has been demonstrated that multiple *Bartonella* spp. (*B. bacilliformis*, *B. quintana*, *B. henselae*, and three *Bartonella vinsonii* subsp. *berkhoffii* genotypes) can induce the *in vitro* production of VEGF.[23–25] *Bartonella* spp. can cause endothelial proliferative disorders, including bacillary angiomatosis and peliosis hepatis, in dogs and humans.[26–31] In combination, these observations suggest the potential for involvement of intra-erythrocytic and endotheliotropic *Bartonella* spp. in the initiation and/or progression of vascular endothelial neoplasia in dogs.

However, in our previous case control study demonstrating an association between *Bartonella* spp. infection and HSA,[22] samples were restricted to a single geographical region (North Carolina) and a single anatomical site (splenic HSA). Seroprevalence studies show that *Bartonella* spp. exposure in dogs can be seen throughout the United States, and there are relatively small but statistically significant regional differences in seroprevalence.[32,33] Additionally, the presence of *Bartonella* spp. DNA in splenic tissue could potentially by explained by the spleen's role in removal of hemotropic parasites from systemic circulation, or by *Bartonella* spp. bacteremia at the time of splenic specimen collection. To address these outstanding questions, this study sought to further clarify the potential involvement of *Bartonella* spp. in HSA in a broader anatomic and geographic context.

The objective of this study was to determine the prevalence of *Bartonella* spp. in conjunction with two other hemotropic pathogens, *Babesia* spp. and hemotropic *Mycoplasma* spp., in tissues and blood samples from dogs with histopathologically diagnosed HSA from throughout the United States. Our hypotheses were: 1) the prevalence of *Bartonella* spp. infection in dogs with HSA would be greater than the prevalence of *Babesia* or hemotropic *Mycoplasma* spp., and 2) the prevalence of *Bartonella* spp. infection in dogs with HSA in the spleen will be similar to prevalence in dogs with HSA in other anatomic locations, such as cardiac muscle.

Methods

Study design and sample sources

This was a retrospective, observational, descriptive study of 110 dogs with HSA. Specimens used for this study were previously collected and banked by the Canine Comparative Oncology and Genomics Consortium (CCOGC) based on previously published standard operating procedures.[34]. Briefly, the CCOGC collected samples from eight participating veterinary university teaching hospitals starting in 2006 with the goal of creating a repository of clinical samples from dogs with common naturally occurring cancers. Prior to sample submission to CCOGC, dogs were given a definitive diagnosis of neoplasia based on histopathology performed by a board-certified veterinary pathologist at the diagnostic laboratory of each participating university. Tissue samples for the CCOGC biospecimen repository were obtained from the primary tumor via surgical biopsy or post-mortem collection (HSA tumor tissue). As an internal control, tissue samples from each dog were also obtained from adjacent grossly normal tissue in

the same organ, or if no grossly normal tissue in the affected organ was apparent, skin biopsies were obtained (non-tumor tissue). Tissue submission (HSA tumor and non-tumor) from each dog required provision of both formalin-fixed (subsequently stored in ethanol) and non-fixed specimens snap-frozen in liquid nitrogen (subsequently stored at -80˚ C). Dogs also had serum and whole blood collected at the time of tissue sampling for submission to the CCOGC.

For this study, samples from dogs diagnosed with HSA were provided by the CCOGC repository to the investigators. Four sample types were provided for pathogen testing: fresh frozen HSA tumor tissue, fresh frozen non-tumor tissue, whole blood, and serum. Two sample types were provided for histopathological confirmation of tissue type and tumor presence included: formalin-fixed HSA tumor tissue and formalin-fixed non-tumor tissue. Any dog that had a diagnosis of HSA (as determined by the CCOGC SOP), and had an adequate amount of fresh frozen tissue stored in the biorepository at the time of the investigators' request for specimens, was included. This study, therefore, included 110 dogs with samples collected between May 2008 and November 2011.

Because of previous sample requests or lack of submission of all requested samples to the CCOGC, there were samples missing when provided to the investigators. Of the 110 dogs, 91 had a complete set of the 4 sample types for pathogen testing (fresh frozen HSA tumor tissue, fresh frozen non-tumor tissue, whole blood, serum) provided to the investigators. No dog was missing more than one sample type, and all dogs had at least one fresh-frozen tissue sample (HSA tumor, non-tumor tissue, or both) available for testing. For this reason, dogs with missing samples for pathogen testing were not excluded from the study. Similarly, of the 110 dogs only 37 had a complete set of the 2 sample types for histopathological confirmation (formalin-fixed HSA tumor tissue, formalin-fixed non-tumor tissue) provided to the investigators. Of the 110 dogs, 37 had formalin-fixed HSA tumor tissue and 93 had formalin-fixed non-tumor tissue provided. There were no formalin-fixed samples of HSA tumor or non-tumor tissue from cardiac tumors available for independent histopathologic review. Because a large proportion of dogs were missing formalin-fixed samples for independent histopathological confirmation, the subgroups of tissues with independent histopathologic confirmation of tissue type and tumor presence was analyzed separately.

The CCOGC provided demographic information for each dog, including age (years), breed, weight (kg) and sex and neuter status. The date and geographic location (university teaching hospital) of sample collection was also provided. The anatomic location of tumor and non-tumor tissue samples for each dog was also provided.

Independent confirmation of demographic and histologic data

When possible, information provided by the CCOGC was independently confirmed by the investigators for this study. Histopathology reports providing the diagnosis of hemangiosarcoma were provided for 102 dogs; these were reviewed by one author (EL) to confirm hemangiosarcoma diagnosis and tumor location.

For dogs with formalin-fixed biopsy samples provided (see above), biopsies were independently evaluated by a board-certified veterinary pathologist (KL) to confirm the tissue and tumor origin of the biopsy. Formalin fixed tissues were submitted to the NCSU College of Veterinary Medicine histology laboratory (Raleigh NC) and tissues were embedded in paraffin blocks (FFPE blocks). Slides containing 5 um sections were prepared from the FFPE blocks and stained with hematoxylin and eosin (H&E). Samples were categorized by organ type and HSA tumor or non-tumor tissue based on H&E staining. If the tissue of origin was not able to be determined from the biopsy (5 tumor samples and 2 non-tumor tissues), or if no formalin-fixed sample was provided (68 tumor samples and 14 non-tumor tissues), the tissue of origin

was categorized by the information provided by the CCOGC and the original diagnostic histopathology reports. Non-tumor tissues from skin or various subcutaneous tissues (including hair, skin, adipose, skeletal muscle, or mammary gland, or any combination of these tissues) were categorized as skin/SQ for analysis.

Pathogen detection methods

DNA was extracted from EDTA anti-coagulated blood and fresh frozen tissue samples using a Qiagen DNeasy® Blood and Tissue kit (Qiagen, Valencia, CA) following the manufacturer's protocols. For each tissue sample (HSA tumor and non-tumor), a 25 mg piece of tissue was excised from the entire sample using a new, prepackaged sterile scalpel. DNA yield and quality was assessed by spectrophotometry (Nanodrop, Wilmington, DE).

Each DNA sample was screened for the presence of *Bartonella* spp. DNA using conventional and qPCR, and *Babesia* spp. and hemotropic *Mycoplasma* spp. using qPCR. *Bartonella* qPCR was performed using primers targeting the 16S-23S intragenic transcribed spacer (ITS) region of *Bartonella* species as described previously[35], in conjunction with a BsppITS438 FAM-labeled hydrolysis probe (TaqMan, Applied Biosystems, Foster City, CA, USA). *Bartonella* qPCR was performed at two dilutions for each sample (using 1 uL and 5 uL of template DNA respectively). PCR screening for *Babesia* spp. and hemotropic *Mycoplasma* spp. were carried out as described previously.[22] Briefly, oligonucleotides Myco16S-322s (`5′ GCCCA TATTCCTACGGGAAGCAGCAGT 3′`) and Myco16S-938as (`5′ CTCCACCACTTGTTCAGGT CCCCGTC 3′`) were used as forward and reverse primers respectively for hemotropic *Mycoplasma* spp. DNA amplification. Oligonucleotides Piro18S-144s (`5′ GATAACCGTGSTAATT STAGGGCTAATACATG 3′`) and Piroplasma18S-722as (`5′ GAATGCCCCCAACCGTTCCTA TTAAC 3′`) were used as forward and reverse primers respectively for *Babesia* spp. DNA amplification.

Amplification was performed in a 25-μl final volume reaction containing 12.5 μl of MyTaq Premix (Bioline), 0.2 μl of 100 μM of each forward primer, reverse primer (IDT® DNA Technology), 7.1 μl of molecular–grade water, and 5 μl of DNA from each sample tested. PCR negative controls were prepared using 5 μl of DNA from blood of a healthy dog. Positive controls for PCR were prepared by using 5 μl of DNA from previously characterized positive dog (clinical cases). Conventional PCR was performed in an Eppendorf Mastercycler EPgradient® under the following conditions: a single hot-start cycle at 95˚C for 2 minutes followed by 55 cycles of denaturing at 94˚C for 15 seconds, annealing at 68˚C for 15 seconds, and extension at 72˚C for 18 seconds. Amplification was completed by an additional cycle at 72˚C for 1 minute, and products were analyzed by 2% agarose gel electrophoresis with detection using ethidium bromide under ultraviolet light.

Validation of positive results was performed by Sanger sequencing of amplicons followed by chromatogram evaluation and sequence alignment using Contig-Express and Align X software (Vector NTI Suite 10.1, Invitrogen Corp, CA, USA). For bacterial species identification, DNA sequences were analyzed for nucleotide sequence homology at NCBI nucleotide database using BLAST version 2.0. A sample was considered *Bartonella* spp. PCR positive if one or more PCR tests (qPCR or conventional PCR) were positive (tests run in parallel). Stringent processing methods were used to avoid DNA carryover during tissue processing.[36] Specifically, tissue samples were processed independently using manual DNA extraction. For all batches of DNA extractions, between 2 and 4 blanks samples (water) were used as negative controls. All negative controls for DNA extractions rendered negative results on all PCR assays. DNA carryover after PCR amplification was avoided by processing each sample in three separate laboratory rooms (one for sample sorting and DNA extraction, a second for

PCR processing, and a third for PCR analysis post amplification), and strict use of personal protective equipment for sample handling by laboratory personnel.

For IFA testing, *Bartonella* antibodies were determined using 3 cell culture grown *Bartonella* spp. (*Bartonella henselae*, *Bartonella vinsonii* subsp. *berkhoffii*, and *Bartonella koehlerae*) as antigens and following standard immunofluorescent antibody assay (IFA) techniques. [37,38] Briefly, bacterial colony isolates were passed from agar plate grown cultures into permissive cell lines. For each antigen, heavily infected cell cultures were spotted onto 30-well Teflon-coated slides (Cel-Line/Thermo Scientific), air-dried, acetone-fixed, and stored frozen. Fluorescein conjugated goat anti-dog IgG (KPL, SeraCare, Milford MA) was used to detect bacteria within cells using a fluorescent microscope (Carl Zeiss Microscopy, LLC, Thornwood NY). Serum samples were diluted in phosphate-buffered saline (PBS) solution containing normal goat serum, Tween-20, and powdered nonfat dry milk to block nonspecific antigen binding sites. Sera were first screened at dilutions of 1:16 to 1:64. All sera that are reactive at 1:64 were further tested with two-fold dilutions to 1:8192.

Study size

An initial power calculation was based on a request for samples from 150 HSA cases from the CCOGC. With 150 HSA cases, assuming the prevalence of *Bartonella* PCR positive cases reported previously in dogs with HSA,[22] using an alpha of 0.05 this study would have 80% power to detect a difference in the prevalence of *Bartonella* spp. DNA in canine cardiac vs. splenic HSA (assuming samples were split evenly between the two tumor locations) if 49% or more, or 7% or less of the cardiac samples were *Bartonella* PCR positive. In addition, assuming the prevalence of each hemotropic pathogen reported previously in dogs with HSA,[22] using an alpha of 0.05 this study would have 80% power to detect similar size differences with 124 cases for hemotropic *Mycoplasma* and 80 cases for *Babesia* spp.

Statistical methods

Descriptive statistics were obtained for demographic factors (age, weight, sex, breed, season of sample collection, geographic location). We assessed statistical differences in the proportion of dogs PCR positive for each hemotropic pathogen using the Chi-squared test of independence (or Fisher's exact tests for small sample numbers). We also assessed statistical differences in the proportion of samples PCR positive from each anatomic location using the Chi-squared test of independence (or Fisher's exact tests for small sample numbers). Differences between demographic factors for *Bartonella* PCR positive and negative dogs were determined using the Wilcoxon Rank-Sum test for continuous variables (age, weight), and Fisher's exact tests for categorical variables. Multivariable logistic regression was used to identify associations between *Bartonella* infection and anatomic location of tissue sample, with potential demographic confounders included as explanatory variables (age, weight, sex, breed, season of sample collection, and geographic location of sample collection). Odds ratios (adjusted ORs) and 95% confidence intervals (95% CIs) were calculated. To determine agreement between tests on two different samples within the same dog, the kappa statistic was calculated.[39] Data analysis was performed using R 3.6.0 (https://www.R-project.org/).

Results

Demographic characteristics of the included dogs are shown in Table 1. There were 58 female dogs (93% spayed) and 52 male dogs (85% neutered). The most common breeds were mixed breed dogs (n = 31), Labrador retrievers (18), and golden retrievers (13); breeds with 3 or fewer individuals were grouped into the Other breed category (34). The median age was 10

Table 1. Demographic and clinicopathologic characteristics of study population. Median and range shown for continuous data. The * indicates a proportion significantly different from baseline (breed baseline = mixed breed).

	All		*Bartonella* +		*Bartonella* -		*p-value*
	Median	Range	Median	Range	Median	Range	
Age (yrs)	10	4–20	10	4–20	10	6–13	0.438
Weight (kg)	33.2	6.1–68.0	34.0	8.5–68.0	30.3	6.1–48.8	0.800
	**All dogs** Number in each category (%)		**Number *Bartonella* + (%)**				**p-value**
Sex							0.31
FI	4 (4)		3 (75)				
FS	54 (49)		37 (69)				
MI	8 (7)		8 (100)				
MN	44 (40)		32 (73)				
Breed							0.046
Mix	31 (28)		26 (84)				
Lab	18 (16)		10 (56)*				
Golden	13 (12)		9 (69)				
GSD	6 (5)		2 (33)*				
Boxer	4 (4)		4 (100)				
Bichon Frise	4 (4)		2 (50)				
Other	34 (31)		27 (79)				
Season							0.778
Autumn	38 (35)		27 (71)				
Winter	23 (21)		18 (78)				
Spring	20 (18)		13 (65)				
Summer	29 (26)		22 (76)				
Location							0.658
MA	65		44 (71)				
WI	19		16 (84)				
CA	9		6 (86)				
MI	6		3 (50)				
MO	3		2 (67)				
OH	3		2 (67)				
TN	3		3 (100)				
CO	2		2 (100)				

https://doi.org/10.1371/journal.pone.0227234.t001

years old, and the median weight was 33.2 kg. No weight was provided for 14 dogs. HSA was diagnosed in all four seasons. Most samples were collected at Tufts University (n = 65) and the University of Wisconsin (19), with between 2–9 dogs included from six other participating veterinary schools (Fig 2).

The number of samples of each type (fresh frozen HSA tumor tissue, fresh frozen non-tumor tissue, whole blood, serum) provided by the CCOGC is shown in Table 2. Of the 100 dogs with fresh frozen HSA tumor samples submitted, 74 were splenic, 14 were cardiac, and 12 were from other sites (5 liver, 2 SQ, 1 lung, 1 undetermined retroperitoneal, 1 undetermined intrathoracic, and 2 undetermined). Of the 104 dogs with fresh frozen non-tumor tissue samples submitted, 39 were splenic, 14 were cardiac, 49 were skin or various subcutaneous tissues (skin/SQ), and 2 were from other sites (1 liver, 1 kidney). The anatomic location of HSA tumor and non-tumor tissue samples is summarized in Table 2. For dogs with splenic HSA tumors, 43% (32/74) had adjacent non-tumor splenic tissue submitted and 49% (36/74) had

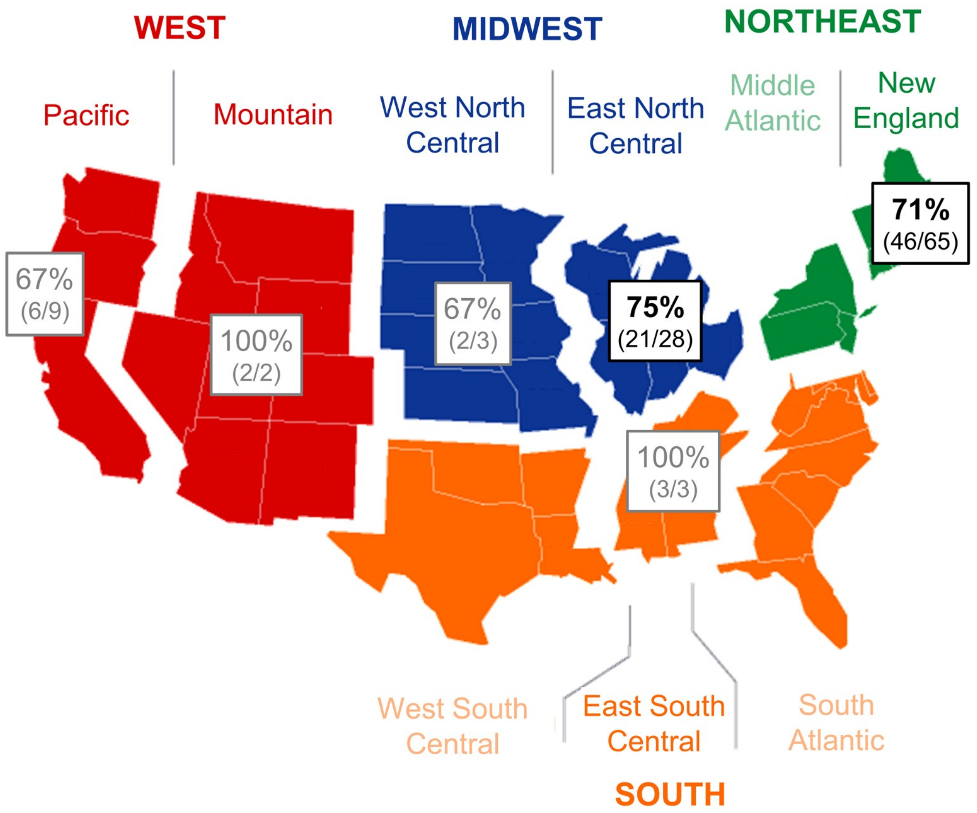

Fig 2. Geographic distribution of dogs with HSA. Shows the percentage of dogs positive for *Bartonella* DNA on PCR of HSA tumor tissue and/or non-tumor tissue biopsy. Color indicates region. Black text indicates regions from which 10 or more samples were received, and gray text indicates regions from < 10 samples were received.

https://doi.org/10.1371/journal.pone.0227234.g002

skin/SQ non-tumor tissue submitted (the remaining 6 dogs with splenic HSA tumors did not have non-tumor tissue submitted). For dogs with HSA tumors from other anatomic locations (including cardiac), the non-tumor tissues submitted were all from the affected organ.

Hemotropic pathogen testing results for all samples are summarized in Table 2. Of the 110 HSA dogs, 80 (73%) were *Bartonella* spp. PCR positive in at least one fresh frozen tissue sample. No dog was *Bartonella* spp. PCR positive on whole blood and only 6 (6%) were IFA seroreactive to *B. henselae*, *B. vinsonii* subsp. *berkhoffii*, or *B. koehlerae* antigens (Fig 3A). In these 6 dogs, 3 were only seroreactive to *B. henselae*, 2 were *B. henselae* and *B. koehlerae* seroreactive, and 1 was only *B. koehlerae* seroreactive.

With the exception of breed, there was no difference in the proportion of dogs *Bartonella* spp. PCR positive based on any of the demographic factors considered (Table 1). The proportion of German Shepherd Dogs (OR 0.60, 95% CI 0.41–0.88) and Labrador retrievers (OR 0.75, 95% CI 0.59–0.97) positive for *Bartonella* spp. DNA was significantly lower than that of mixed breed dogs. When adjusted for other potentially confounding demographic variables using multivariable logistic regression, only Labrador retrievers had a lower proportion positive for *Bartonella* spp. DNA compared to mixed breed dogs (OR 0.69, 95% CI 0.52–0.92). The proportion of *Bartonella* spp. PCR positive dogs from each geographic location, based on the location of the submitting veterinary college, is shown in Fig 2. Tthere were no statistically significant differences in *Bartonella* spp. between geographic locations (Table 1), with *Bartonella* positive dogs distributed broadly throughout the United States.

Table 2. Anatomic location of samples. Number of samples tested from each anatomic location, and number of each sample positive for each pathogen tested. Serum was tested for *Bartonella* spp. antibodies using IFA; serology for hemotropic *Mycoplasma* and *Babesia* spp. was not performed. All other samples were tested by PCR for DNA of each pathogen.

	Number of samples	Number of samples (%) *Bartonella* spp. positive	Subgroup of independently confirmed samples: # *Bartonella* spp positive/# tested (% *Bartonella* spp. positive)	Number of samples hemotropic *Mycoplasma* spp. positive	Number of samples *Babesia* spp. positive
HSA tumors					
Spleen	74	24 (32)	5/18 (28%)	1	0
Cardiac	14	8 (57)	0/0 (N/A)	0	0
Other	12	2 (17)	0/3 (0%)	0	0
TOTAL	100	34 (34)		1	0
Non-tumor tissue					
Spleen	39	22 (56)	19/35 (54%)	0	0
Cardiac	14	13 (93)	0/0 (N/A)	1	0
Skin/SQ	49	31 (63)	31/49 (63%)	1	0
Other	2	0 (0)	0/2 (0%)	0	0
TOTAL	104	66 (63)		2	0
Blood					
Whole blood	108	0	NA	2	0
Serum	107	6	NA	N/A	N/A

https://doi.org/10.1371/journal.pone.0227234.t002

Fresh frozen tissues from only 3 dogs were hemotropic *Mycoplasma* spp. PCR positive, a significantly smaller proportion (3%, $p < 0.0001$) compared to *Bartonella* spp. (Fig 4). Hemotropic *Mycoplasma* spp. DNA was amplified from two non-tumor tissues (one skin/SQ and one cardiac), and one HSA tumor (spleen). The 2 hemotropic *Mycoplasma* spp. positive non-tumor tissues were both also *Bartonella* spp. PCR positive; the hemotropic *Mycoplasma* positive tumor tissues was *Bartonella* spp. PCR negative. Hemotropic *Mycoplasma* spp. DNA was amplified from whole blood of two dogs, neither of which had hemotropic *Mycoplasma* spp. DNA in their tissues. *Babesia* spp. DNA was not amplified from any whole blood or tissue specimen (Fig 4).

The proportion of HSA tumor and non-tumor tissues *Bartonella* spp. PCR positive for each anatomic location is summarized in Fig 3B. The proportion of non-tumor tissues that were *Bartonella* spp. PCR positive (63%) was significantly higher than the proportion of HSA tumors that were *Bartonella* spp. PCR positive 34%, ($p < 0.001$). The proportion of *Bartonella* spp. PCR positive HSA tumor samples did not differ significantly between anatomic location ($p = 0.083$). Based on the multivariable logistic regression model, the anatomic location of the HSA tumor was not associated with *Bartonella* spp. PCR positivity. The proportions of non-tumor tissues *Bartonella* spp. PCR positive were significantly different based on anatomic location ($p < 0.0001$), with a higher proportion of non-tumor cardiac samples positive for *Bartonella* spp. PCR compared to non-tumor spleen samples when adjusted for possible demographic confounders (adjusted OR 1.75, 95% CI 1.25–2.47).

When comparing the *Bartonella* spp. PCR results for HSA tumor and non-tumor tissues, there was only slight agreement between the two samples (kappa = 0.14). Only 36% of dogs with a *Bartonella* spp. PCR positive non-tumor tissue sample had a positive HSA tumor, whereas 76% of dogs with a positive HSA tumor sample were positive in non-tumor tissue.

In both tumor and non-tumor samples, the most common *Bartonella* species identified was *B. henselae*. Homologies ranged from 99.3% to 100% (of 138 bp analyzed) with *B. henselae*

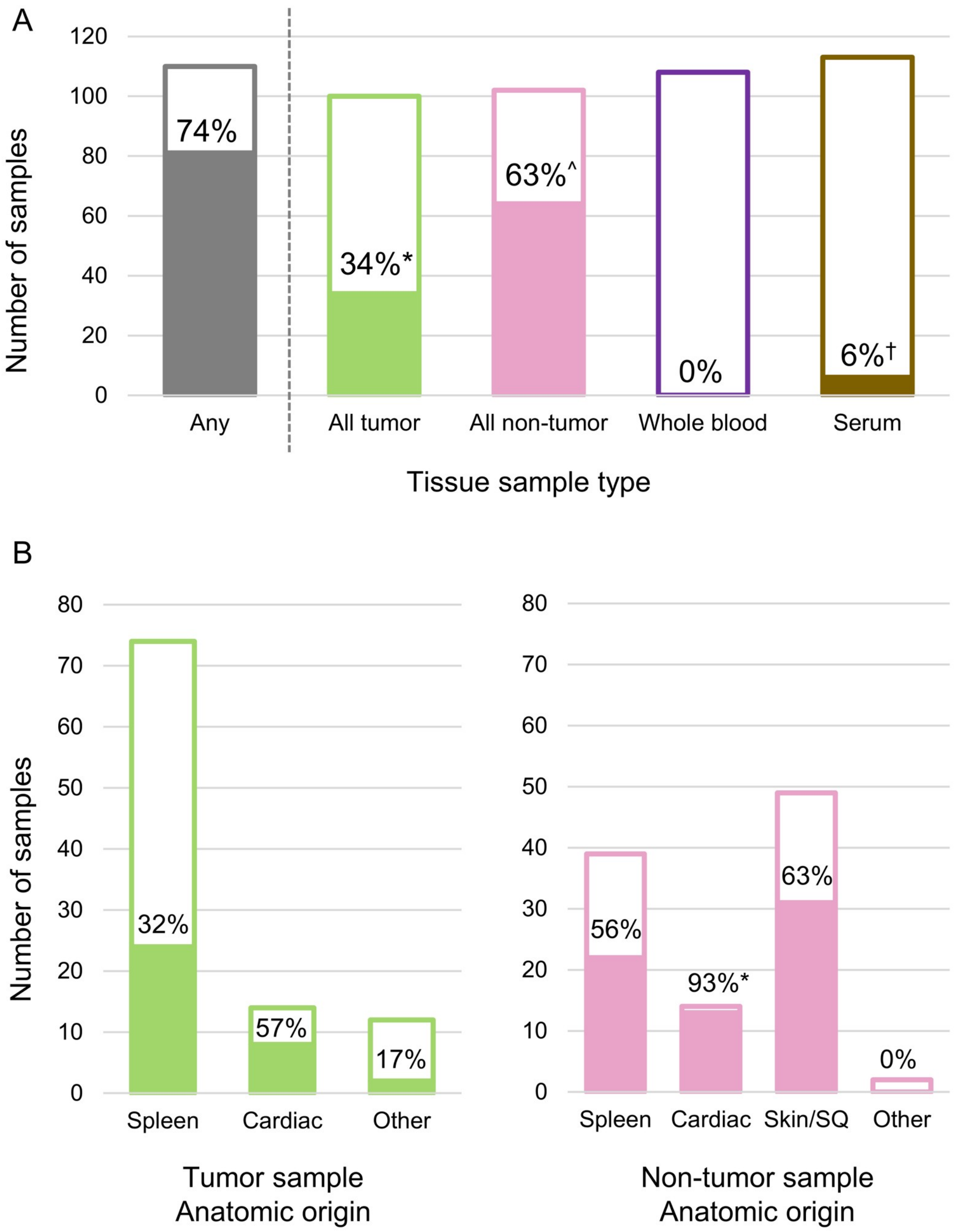

Fig 3. Proportion of samples positive for *Bartonella* spp. Color indicates sample type (HSA tumors, green; non-tumor tissue, pink; whole blood, purple; serum, brown; any sample, grey). A) Blood, serum, HSA tumors, and non-tumor tissues (all anatomic locations combined). Blood and tissue were tested by PCR for *Bartonella* spp. DNA, serum was tested by IFA for *Bartonella* spp. antibodies. Different superscripts indicate significantly different proportions ($p < 0.05$, Chi-squared test) B) Left panel shows HSA tumors by anatomic location, right panel shows non-tumor tissues by anatomic location. The * indicates a statistically significant difference in proportion from spleen samples ($p < 0.05$, Chi-squared or Fisher's exact test).

https://doi.org/10.1371/journal.pone.0227234.g003

CAL1 and SA2 (Genbank accessions AF369527and AF369529, respectively). Of the HSA tumors, 27 contained *B. henselae* (including 2 co-infected with *B. henselae* and *B. koehlerae*), 1 contained only *B. koehlerae* (140/140 bp, 100% homology with Genbank accession AF312490), 1 contained *Bartonella apis* (94/94 bp, 100% homology with Genbank accession CP015625)

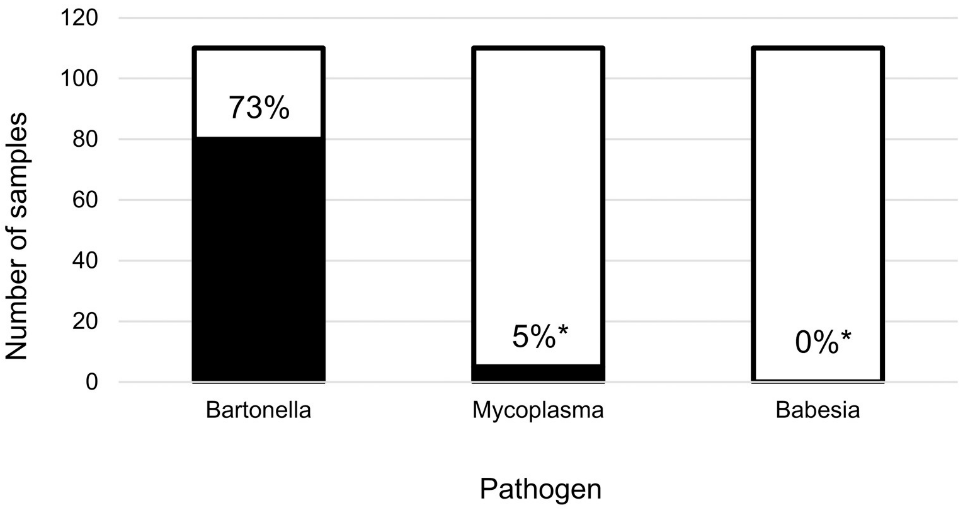

Fig 4. Proportion of dogs with HSA positive for hemotropic pathogen DNA on any sample. * indicates statistically significant difference in proportion from *Bartonella* spp. ($p < 0.05$, Chi-squared test).

https://doi.org/10.1371/journal.pone.0227234.g004

and 3 had a *Bartonella* species that was most closely related to *B*. *henselae* (with homology ranging from 95% to 98% with either *B*. *henselae* CAL1 or SA2). In non-tumor tissues, 58 contained *B*. *henselae*, 1 contained *B*. *koehelerae* (140/140 bp, 100% homology with Genbank accession AF312490), 6 contained a *Bartonella* species that was most closely related to *B*. *henselae* (with homology ranging from 95% to 98% with either *B*. *henselae* CAL1 or SA2), and 1 contained a *Bartonella* species that was unable to be identified to the species level.

When considering only those tissue samples that were able to be independently assigned an anatomic location and tumor presence based on histopathology of formalin-fixed samples performed by the investigators, the proportions of each tissue type positive for *Bartonella* spp. DNA by PCR (Table 2) was similar to that of the entire sample set. For HSA tumors in the spleen, the independently confirmed subgroup had 5 of 18 positive (28%), compared to the entire set with 32% positive ($p = 0.515$). For non-tumor tissues from the spleen, the independently confirmed subgroup had 19 of 35 positive (56%), compared to the entire set with 54% positive ($p = 0.960$).

Discussion

Overall, in this study 73% of dogs with HSA were *Bartonella* spp. PCR positive on HSA tumor tissue, non-tumor tissue, or bothtissue samples. Consistent with our previous study,[22] the proportion of *Bartonella* spp. infection in dogs with HSA was significantly greater than the proportion of *Babesia* or hemotropic *Mycoplasma* spp. Somewhat surprisingly, *Bartonella* spp. DNA was amplified more often from non-tumor tissues (63%) compared to HSA tumors tissues (34%).

There are several potential explanations for the difference in *Bartonella* prevalence between HSA tumor and non-tumor tissues. It is possible that in large vascular splenic tumors there are necrotic or infarcted regions that contain fewer organisms, placing these samples below the level of PCR detection. This possibility is supported by amplification of *Bartonella* spp. DNA from 75% of non-tumor tissues obtained from the 32 dogs that were PCR positive from their HSA tumors. It is also possible that the high prevalence of *Bartonella* DNA in non-tumor tissues reflects asymptomatic carriage and is not associated with HSA. However since all 110 dogs had HSA, the higher prevalence in non-tumor tissue does not necessarily contradict the

hypothesis that *Bartonella* infection is associated with HSA–rather, in the absence of a control group of dogs without HSA, we cannot conclude whether this high prevalence is seen in the absence of HSA as well. Further studies using a case-control or cohort design to compare *Bartonella* spp. PCR prevalence between dogs with HSA and without HSA will be needed to determine if this is the case.

While the proportion of dogs with *Bartonella* spp. DNA in one or more tissue samples was surprisingly high, no dog had *Bartonella* DNA amplified from a blood sample. This suggests that *Bartonella* DNA found in any given tissue is not due to blood contamination of the tissue, but rather that the *Bartonella* may be intracellularly localized within that tissue. Similar results have been reported previously: in one case dogs experimentally infected with *Bartonella* spp. failed to become detectably bacteremic, despite *Bartonella* spp. being isolated from tissues (bone marrow and lung) post-mortem; in another, a dog had *B. henselae* DNA amplified from biopsies of vasculitis lesions and normal skin, but no *Bartonella* DNA on multiple sequential blood samples.[40,41] Reasons that *Bartonella* DNA is not found in blood samples from dogs with *Bartonella* present in tissue could include intermittent bacteremia, rapid clearance of bacteremia by the dogs' immune system, or low levels of bacteremia that are below the current limit of PCR detection. Regardless, *Bartonella* blood PCR does not effectively predict whether a dog is infected with a *Bartonella* spp. Similarly, *Bartonella* spp. serology was also positive in only a small fraction of those dogs with positive *Bartonella* spp. PCR in tissue. Previous results documenting poor agreement between *Bartonella* spp. exposure based on IFA, and *Bartonella* spp. bacteremia in dogs have been reported.[42,43] Finally, in comparison with FFPE splenic HSA tumors tested for *Bartonella* in our previous study, *Bartonella* testing on fresh-frozen splenic HSA tumors yielded very slightly higher levels of detection (26% of fixed tissue vs. 28–32% of fresh frozen tissue). Based upon this study, we recommend that when possible, fresh frozen tissue biopsies from either the affected organ, or unaffected skin, be collected and submitted for PCR testing to maximize the potential for detection of *Bartonella* spp. Future studies will be needed to guide medical decision making when *Bartonella* spp. infection is documented in a dog with HSA.

To address the possibility that *Bartonella* spp. DNA is found in high proportions of dogs' spleens due to the spleen's role in immunologic clearance of bacteria or infected erythrocytes from circulation, we tested multiple other organs for the presence of *Bartonella* DNA. Rather than seeing the highest proportions of splenic tissue positive for *Bartonella* DNA, in both tumor and non-tumor tissues we found cardiac tissues to have a higher proportion positive. This may reflect a tissue tropism of *Bartonella* for cardiac tissue, which would be compatible with the known ability of *Bartonella* to infect heart valves as a cause of endocarditis (or less commonly, myocarditis).[44–47] Additionally, when examining non-tumor tissues, we found a similar proportion of skin/SQ samples were positive for *Bartonella* compared to spleen samples. This may reflect an alternative tissue tropism for the bacteria, which is vector-borne and relies on the bite of an arthropod vector for transmission. Establishing latent infection in the skin could be an evolutionary response to enhance the likelihood of uptake during blood feeding by arthropod vectors. Based on the results presented here, it is unlikely that the high proportion of dog spleen samples positive for *Bartonella* spp. DNA on PCR reflect solely splenic clearance of bacteria or infected erythrocytes.

Because of the surprisingly high proportion of dogs with HSA with *Bartonella* DNA in tissue samples in this study, and the well-established ability of *Bartonella* spp. to induce angiogenesis and chronic inflammation *in vivo* and *in vitro*, we speculate that *B. henselae* could be a cause or cofactor in the development of HSA in dogs. Angiogenesis is a fundamental component of primary tumor cell proliferation and metastasis, particularly in HSA.[21,48–52] Specifically, dogs with HSA have increased amounts of plasma VEGF compared to healthy dogs,[48]

VEGF and its receptors are present in tumors (when evaluated with IHC)[52] and upregulated in HSA compared to benign hemangioma[49] (and in xenograft tumors using a mouse model) [50]. *In vitro*, *B. henselae* induces angiogenesis and proliferation of endothelial cells in part by stimulating production of VEGF.[23–25,31,53,54] It is well established that *Bartonella* spp. cause the non-neoplastic endothelial proliferative disorders bacillary angiomatosis and peliosis hepatis in humans, and there have also been rare reports of these conditions in dogs infected with *Bartonella* spp.[26–31,53,55,56] In addition, there have been case reports documenting neoplastic endothelial cell tumors in humans and dogs infected with *Bartonella* spp.: *B. vinsonii* subsp. *berkhoffii* was isolated from a dog with hemangiopericytoma, and from humans residing on three continents with epithelioid hemangioendothelioma.[57,58] The high prevalence of *Bartonella* DNA in tissues from dogs with HSA supports the need for further studies on the mechanistic basis of a potential link between *Bartonella* spp. infection and vascular endothelial cancers like HSA.

Limitations of this study include the use of archived specimens, which precluded systematic sampling from particular organs of interest. This study was designed to be descriptive, and as such there was no control group consisting of dogs without HSA. Because of the lack of control group in this study, we cannot determine whether *Bartonella* spp. infection is epidemiologically associated with HSA. Additionally, only approximately one third of tumor tissue samples were able to be independently reviewed to confirm their anatomic tissue of origin and that they contained neoplastic cells. However, the overall pattern of lower Bartonella DNA prevalence in HSA tumor tissue compared to non-tumor tissue that was evident in both the spleen and cardiac tissues was also present in the subgroup of splenic tissues that did have independent histopathologic confirmation of anatomic location and tumor cell presence. There was not fixed tissue provided for independent histopathologic review from any of the cardiac samples (tumor or non-tumor), so diagnosis relied on the histopathologic reports from the submitting veterinary colleges. This may have led to misclassification of cardiac tissues as tumor or non-tumor. However due to the biological behavior of HSA in the heart, with most tumors presenting as a mass involving the right atrium (even in dogs with concurrent splenic HSA), correct classification of tissue as HSA (mass in right atrium) or non-tumor (other unaffected cardiac tissue) at the time of sample collection was assumed to be accurate. This study was also limited by using IFA to detect *Bartonella* spp. antibodies. IFA is known to have low sensitivity and may underestimate the true seroprevalence of *Bartonella* spp. in dogs.[42,59–62] The use of other serological assays currently under development, such as Western Blot or ELISA, may improve sensitivity of detection of seroreactivity. Finally, the initial power calculations for determining a statistically significant difference in *Bartonella* infection of tumors from different anatomic locations (splenic vs. cardiac) was based on 75 splenic and 75 cardiac cases. In fact, we received many fewer cardiac samples than expected, which decreased the power of this aim of the study. With the sample size used (69 splenic and 13 cardiac), the study was underpowered to detect the empiric difference that was found (33% *Bartonella* PCR positive for splenic and 54% for cardiac). In contrast, because all 105 dogs were able to be tested for each pathogen, despite the slightly smaller sample size than expected this aim was adequately powered to detect the empiric differences found (74% *Bartonella* spp. PCR positive, 3% hemotropic *Mycoplasma* PCR positive, 0% *Babesia* spp. PCR positive).

We conclude that our findings strengthen the need to further investigate the role of *Bartonella* in the development of HSA, particularly with well controlled epidemiologic studies and mechanistic research to identify how this genus may contribute to tumor development. Ultimately, the development of a vaccine to protect dogs against *Bartonella* infection could potentially decrease the prevalence of this highly malignant neoplasm.

Author Contributions

Conceptualization: Keith E. Linder, Matthew Breen, Ricardo G. Maggi, Edward B. Breitschwerdt.

Data curation: Erin Lashnits, Pradeep Neupane, Keith E. Linder, Matthew Breen.

Formal analysis: Erin Lashnits, Keith E. Linder, Ricardo G. Maggi, Edward B. Breitschwerdt.

Funding acquisition: Keith E. Linder, Matthew Breen, Ricardo G. Maggi, Edward B. Breitschwerdt.

Investigation: Pradeep Neupane, Julie M. Bradley, Toni Richardson, Keith E. Linder, Ricardo G. Maggi.

Methodology: Pradeep Neupane, Rachael Thomas, Keith E. Linder, Matthew Breen, Ricardo G. Maggi, Edward B. Breitschwerdt.

Project administration: Julie M. Bradley, Matthew Breen, Edward B. Breitschwerdt.

Resources: Rachael Thomas, Keith E. Linder, Matthew Breen, Ricardo G. Maggi, Edward B. Breitschwerdt.

Supervision: Matthew Breen, Edward B. Breitschwerdt.

Validation: Matthew Breen, Ricardo G. Maggi, Edward B. Breitschwerdt.

Visualization: Erin Lashnits, Keith E. Linder.

Writing – original draft: Erin Lashnits, Toni Richardson, Edward B. Breitschwerdt.

Writing – review & editing: Erin Lashnits, Pradeep Neupane, Julie M. Bradley, Rachael Thomas, Ricardo G. Maggi, Edward B. Breitschwerdt.

References

1. Knoll LJ, Hogan DA, Leong JM, Heitman J, Condit RC. Pearls collections: What we can learn about infectious disease and cancer. PLOS Pathog. 2018 Mar 29; 14(3):e1006915. https://doi.org/10.1371/journal.ppat.1006915 PMID: 29596508
2. Plummer M, de Martel C, Vignat J, Ferlay J, Bray F, Franceschi S. Global burden of cancers attributable to infections in 2012: a synthetic analysis. Lancet Glob Heal. 2016 Sep 1; 4(9):e609–16.
3. Group IA for R on CW. Biological Agents: A review of human carcinogens. Vol. 100B, IARC Monographs on the Evaluation of Carcinogenic Risks to Humans. Geneva, Switzerland; 2012.
4. Riley DR, Sieber KB, Robinson KM, White JR, Ganesan A, Nourbakhsh S, et al. Bacteria-Human Somatic Cell Lateral Gene Transfer Is Enriched in Cancer Samples. PLoS Comput Biol. 2013; 9(6).
5. Lax AJ, Thomas W, Kuper H, Parsonnet J, et al. How bacteria could cause cancer: one step at a time. Trends Microbiol. 2002 Jun; 10(6):293–9. https://doi.org/10.1016/s0966-842x(02)02360-0 PMID: 12088666
6. Molnar B, Galamb O, Sipos F, Leiszter K, Tulassay Z. Molecular Pathogenesis of Helicobacter pylori Infection: The Role of Bacterial Virulence Factors. Dig Dis. 2010; 28(4–5):604–8. https://doi.org/10.1159/000320060 PMID: 21088410
7. Pagano JS, Blaser M, Buendia M-A, Damania B, Khalili K, Raab-Traub N, et al. Infectious agents and cancer: criteria for a causal relation. Semin Cancer Biol. 2004 Dec; 14(6):453–71. https://doi.org/10.1016/j.semcancer.2004.06.009 PMID: 15489139
8. Prymak C, McKee LJ, Goldschmidt MH, Glickman LT. Epidemiologic, clinical, pathologic, and prognostic characteristics of splenic hemangiosarcoma and splenic hematoma in dogs: 217 cases (1985). J Am Vet Med Assoc. 1988 Sep 15; 193(6):706–12. PMID: 3192450
9. Mullin C, Clifford CA. Histiocytic Sarcoma and Hemangiosarcoma Update. Vet Clin North Am Small Anim Pract. 2019 Jun 8;
10. Spangler WL, Culbertson MR. Prevalence, type, and importance of splenic diseases in dogs: 1,480 cases (1985–1989). J Am Vet Med Assoc. 1992 Mar 15; 200(6):829–34. PMID: 1568933

11. Schultheiss PC. A Retrospective Study of Visceral and Nonvisceral Hemangiosarcoma and Hemangiomas in Domestic Animals. J Vet Diagnostic Investig. 2004 Nov 25; 16(6):522–6.
12. Grüntzig K, Graf R, Boo G, Guscetti F, Hässig M, Axhausen KW, et al. Swiss Canine Cancer Registry 1955–2008: Occurrence of the Most Common Tumour Diagnoses and Influence of Age, Breed, Body Size, Sex and Neutering Status on Tumour Development. J Comp Pathol. 2016 Aug; 155(2–3):156–70. https://doi.org/10.1016/j.jcpa.2016.05.011 PMID: 27406312
13. Hart BL, Hart LA, Thigpen AP, Willits NH. Neutering of German Shepherd Dogs: associated joint disorders, cancers and urinary incontinence. Vet Med Sci. 2016 Aug; 2(3):191–9. https://doi.org/10.1002/vms3.34 PMID: 29067194
14. Kent MS, Burton JH, Dank G, Bannasch DL, Rebhun RB. Association of cancer-related mortality, age and gonadectomy in golden retriever dogs at a veterinary academic center (1989–2016). Bauer JA, editor. PLoS One. 2018 Feb 6; 13(2):e0192578. https://doi.org/10.1371/journal.pone.0192578 PMID: 29408871
15. Treggiari E, Pedro B, Dukes-McEwan J, Gelzer AR, Blackwood L. A descriptive review of cardiac tumours in dogs and cats. Vet Comp Oncol. 2017 Jun; 15(2):273–88. https://doi.org/10.1111/vco.12167 PMID: 26420436
16. Dana M. ONLINE CASE REPORTS Pericardial Hemangiosarcoma in a 10-Year-Old Papillon.
17. Johnson KA, Powers BE, Withrow SJ, Sheetz MJ, Curtis CR, Wrigley RH. Splenomegaly in dogs. Predictors of neoplasia and survival after splenectomy. J Vet Intern Med. 3(3):160–6. https://doi.org/10.1111/j.1939-1676.1989.tb03092.x PMID: 2778749
18. Schick AR, Hayes GM, Singh A, Mathews KG, Higginbotham ML, Sherwood JM. Development and validation of a hemangiosarcoma likelihood prediction model in dogs presenting with spontaneous hemoabdomen: The HeLP score. J Vet Emerg Crit Care. 2019 May 17; 29(3):239–45.
19. Batschinski K, Nobre A, Vargas-Mendez E, Tedardi M V, Cirillo J, Cestari G, et al. Canine visceral hemangiosarcoma treated with surgery alone or surgery and doxorubicin: 37 cases (2005–2014). Can Vet J = La Rev Vet Can. 2018; 59(9):967–72.
20. Moore AS, Rassnick KM, Frimberger AE. Evaluation of clinical and histologic factors associated with survival time in dogs with stage II splenic hemangiosarcoma treated by splenectomy and adjuvant chemotherapy: 30 cases (2011–2014). J Am Vet Med Assoc. 2017 Sep 1; 251(5):559–65. https://doi.org/10.2460/javma.251.5.559 PMID: 28828962
21. Tamburini BA, Phang TL, Fosmire SP, Scott MC, Trapp SC, Duckett MM, et al. Gene expression profiling identifies inflammation and angiogenesis as distinguishing features of canine hemangiosarcoma. BMC Cancer. 2010 Dec 9; 10(1):619.
22. Varanat M, Maggi RG, Linder KE, Breitschwerdt EB. Molecular Prevalence of Bartonella, Babesia, and Hemotropic Mycoplasma sp. in Dogs with Splenic Disease. J Vet Intern Med. 2011; 25(6):1284–91. https://doi.org/10.1111/j.1939-1676.2011.00811.x PMID: 22092618
23. Beerlage C, Varanat M, Linder K, Maggi RG, Cooley J, Kempf VAJ, et al. Bartonella vinsonii subsp. berkhoffii and Bartonella henselae as potential causes of proliferative vascular diseases in animals. Med Microbiol Immunol. 2012 Aug 27; 201(3):319–26. https://doi.org/10.1007/s00430-012-0234-5 PMID: 22450733
24. Kempf VAJ, Volkmann B, Schaller M, Sander CA, Alitalo K, Rieß T, et al. Evidence of a leading role for VEGF in *Bartonella henselae* -induced endothelial cell proliferations. Cell Microbiol. 2001 Sep; 3(9):623–32. https://doi.org/10.1046/j.1462-5822.2001.00144.x PMID: 11553014
25. Devraj G, Beerlage C, Brüne B, Kempf VAJ. Hypoxia and HIF-1 activation in bacterial infections. Microbes Infect. 2017 Mar; 19(3):144–56. https://doi.org/10.1016/j.micinf.2016.11.003 PMID: 27903434
26. Berkowitz ST, Gannon KM, Carberry CA, Cortes Y. Resolution of spontaneous hemoabdomen secondary to peliosis hepatis following surgery and azithromycin treatment in a Bartonella species infected dog. J Vet Emerg Crit Care. 2016; 00(0):1–7.
27. Yager JA, Best SJ, Maggi RG, Varanat M, Znajda N, Breitschwerdt EB. Bacillary angiomatosis in an immunosuppressed dog. Vet Dermatol. 2010 Mar; 21(4):420–8. https://doi.org/10.1111/j.1365-3164.2010.00879.x PMID: 20374571
28. Kostianovsky M. Angiogenic process in bacillary angiomatosis. Ultrastruct Pathol. 1994; 18(3):349. https://doi.org/10.3109/01913129409023203 PMID: 7520641
29. Perkocha LA, Geaghan SM, Yen TS, Nishimura SL, Chan SP, Garcia-Kennedy R, et al. Clinical and pathological features of bacillary peliosis hepatis in association with human immunodeficiency virus infection. Vol. 323, The New England journal of medicine. 1990. p. 1581–6.
30. Anstead GM. The centenary of the discovery of trench fever, an emerging infectious disease of World War 1. Lancet Infect Dis. 2016; 16(8):e164–72. https://doi.org/10.1016/S1473-3099(16)30003-2 PMID: 27375211

31. Berrich M, Kieda C, Grillon C, Monteil M, Lamerant N, Gavard J, et al. Differential effects of bartonella henselae on human and feline macro- and micro-vascular endothelial cells. PLoS One. 2011; 6(5).

32. Lashnits E, Correa M, Hegarty BC, Birkenheuer A, Breitschwerdt EB. Bartonella Seroepidemiology in Dogs from North America, 2008–2014. J Vet Intern Med. 2018 Jan; 32(1):222–31. https://doi.org/10.1111/jvim.14890 PMID: 29197186

33. Yancey CB, Hegarty BC, Qurollo BA, Levy MG, Birkenheuer AJ, Weber DJ, et al. Regional seroreactivity and vector-borne disease co-exposures in dogs in the United States from 2004–2010: utility of canine surveillance. Vector Borne Zoonotic Dis. 2014; 14(10):724–32. https://doi.org/10.1089/vbz.2014.1592 PMID: 25325316

34. Mazcko C, Thomas R. The Establishment of the Pfizer-Canine Comparative Oncology and Genomics Consortium Biospecimen Repository. Vet Sci. 2015 Jul 7; 2(3):127–30. https://doi.org/10.3390/vetsci2030127 PMID: 29061936

35. Breitschwerdt EB, Maggi RG, Duncan AW, Nicholson WL, Hegarty BC, Woods CW. Bartonella Species in Blood of Immunocompetent Persons with Animal and Arthropod Contact. Emerg Infect Dis. 2007 Jun; 13(6):938–41. https://doi.org/10.3201/eid1306.061337 PMID: 17553243

36. Varanat M, Maggi RG, Linder KE, Horton S, Breitschwerdt EB. Cross-contamination in the Molecular Detection of *Bartonella* from Paraffin-embedded Tissues. Vet Pathol. 2009 Sep 9; 46(5):940–4. https://doi.org/10.1354/vp.08-VP-0259-B-BC PMID: 19429988

37. Maggi RG, Birkenheuer AJ, Hegarty BC, Bradley JM, Levy MG, Breitschwerdt EB. Comparison of serological and molecular panels for diagnosis of vector-borne diseases in dogs. Parasit Vectors. 2014; 7(1):127.

38. Maggi RG, Mascarelli PE, Pultorak EL, Hegarty BC, Bradley JM, Mozayeni BR, et al. Bartonella spp. bacteremia in high-risk immunocompetent patients. Diagn Microbiol Infect Dis. 2011; 71(4):430–7. https://doi.org/10.1016/j.diagmicrobio.2011.09.001 PMID: 21996096

39. McHugh ML. Interrater reliability: the kappa statistic. Biochem medica. 2012; 22(3):276–82.

40. Balakrishnan N, Cherry Na, Linder KE, Pierce E, Sontakke N, Hegarty BC, et al. Experimental infection of dogs with Bartonella henselae and Bartonella vinsonii subsp. berkhoffii. Vet Immunol Immunopathol. 2013; 156(1–2):153–8. https://doi.org/10.1016/j.vetimm.2013.09.007 PMID: 24120155

41. Southern BL, Neupane P, Ericson ME, Dencklau JC, Linder KE, Bradley JM, et al. *Bartonella henselae* in a dog with ear tip vasculitis. Vet Dermatol. 2018 Dec; 29(6):537–e180. https://doi.org/10.1111/vde.12695 PMID: 30318847

42. Neupane P, Hegarty BC, Marr HS, Maggi RG, Birkenheuer AJ, Breitschwerdt EB. Evaluation of cell culture-grown *Bartonella* antigens in immunofluorescent antibody assays for the serological diagnosis of bartonellosis in dogs. J Vet Intern Med. 2018 Nov 1; 32(6):1958–64. https://doi.org/10.1111/jvim.15301 PMID: 30307643

43. Pérez Vera C, Diniz PPVP, Pultorak EL, Maggi RG, Breitschwerdt EB. An unmatched case controlled study of clinicopathologic abnormalities in dogs with Bartonella infection. Comp Immunol Microbiol Infect Dis. 2013; 36(5):481–7. https://doi.org/10.1016/j.cimid.2013.04.001 PMID: 23683861

44. Chomel BB, Kasten RW, Williams C, Wey AC, Henn JB, Maggi R, et al. Bartonella endocarditis: a pathology shared by animal reservoirs and patients. Ann N Y Acad Sci. 2009; 1166:120–6. https://doi.org/10.1111/j.1749-6632.2009.04523.x PMID: 19538271

45. MacDonald KA, Chomel BB, Kittleson MD, Kasten RW, Thomas WP, Pesavento P. A Prospective Study of Canine Infective Endocarditis in Northern California (1999–2001): Emergence of Bartonella as a Prevalent Etiologic Agent. J Vet Intern Med. 2004 Jan; 18(1):56–64. https://doi.org/10.1892/0891-6640(2004)18<56:apsoci>2.0.co;2 PMID: 14765733

46. Roura X, Santamarina G, Tabar M-D, Francino O, Altet L. Polymerase chain reaction detection of Bartonella spp. in dogs from Spain with blood culture-negative infectious endocarditis. J Vet Cardiol. 2018 Aug 25; 20(4):267–75. https://doi.org/10.1016/j.jvc.2018.04.006 PMID: 29807750

47. Fenimore A, Varanat M, Maggi R, Schultheiss P, Breitschwerdt E, Lappin MR. Bartonella spp. DNA in cardiac tissues from dogs in colorado and wyoming. J Vet Intern Med. 2011 May; 25(3):613–6. https://doi.org/10.1111/j.1939-1676.2011.0722.x PMID: 21539606

48. Clifford CA, Hughes D, Beal MW, Mackin AJ, Henry CJ, Shofer FS, et al. Plasma Vascular Endothelial Growth Factor Concentrations in Healthy Dogs and Dogs with Hemangiosarcoma. J Vet Intern Med. 2001; 15(2):131. https://doi.org/10.1892/0891-6640(2001)015<0131:pvegfc>2.3.co;2 PMID: 11300596

49. Yonemaru K, Sakai H, Murakami M, Yanai T, Masegi T. Expression of vascular endothelial growth factor, basic fibroblast growth factor, and their receptors (flt-1, flk-1, and flg-1) in canine vascular tumors. Vet Pathol. 2006; 43(6):971–80. https://doi.org/10.1354/vp.43-6-971 PMID: 17099154

50. Kodama A, Sakai H, Matsuura S, Murakami M, Murai A, Mori T, et al. Establishment of canine hemangiosarcoma xenograft models expressing endothelial growth factors, their receptors, and angiogenesis-

associated homeobox genes. BMC Cancer. 2009 Oct 14; 9:363. https://doi.org/10.1186/1471-2407-9-363 PMID: 19825192

51. Abou Asa S, Mori T, Maruo K, Khater A, El-sawak A, Abd el-Aziz E, et al. Analysis of genomic mutation and immunohistochemistry of platelet-derived growth factor receptors in canine vascular tumours. Vet Comp Oncol. 2015 Sep 1; 13(3):237–45. https://doi.org/10.1111/vco.12035 PMID: 23611531
52. Göritz M, Müller K, Krastel D, Staudacher G, Schmidt P, Kühn M, et al. Canine splenic haemangiosarcoma: Influence of metastases, chemotherapy and growth pattern on post-splenectomy survival and expression of angiogenic factors. J Comp Pathol. 2013 Jul; 149(1):30–9. https://doi.org/10.1016/j.jcpa.2012.11.234 PMID: 23276383
53. Pons MJ, Gomes C, Aguilar R, Barrios D, Aguilar-Luis MA, Ruiz J, et al. Immunosuppressive and angiogenic cytokine profile associated with Bartonella bacilliformis infection in post-outbreak and endemic areas of Carrion's disease in Peru. Caimano MJ, editor. PLoS Negl Trop Dis. 2017 Jun 19; 11(6): e0005684. https://doi.org/10.1371/journal.pntd.0005684 PMID: 28628613
54. Scheidegger F, Quebatte M, Mistl C, Dehio C. The Bartonella henselae VirB/Bep system interferes with vascular endothelial growth factor (VEGF) signalling in human vascular endothelial cells. Cell Microbiol. 2011 Mar 1; 13(3):419–31. https://doi.org/10.1111/j.1462-5822.2010.01545.x PMID: 21044238
55. Cerimele F, Brown LF, Bravo F, Ihler GM, Kouadio P, Arbiser JL. Infectious Angiogenesis: Bartonella bacilliformis Infection Results in Endothelial Production of Angiopoetin-2 and Epidermal Production of Vascular Endothelial Growth Factor. Am J Pathol. 2003 Oct 1; 163(4):1321–7. https://doi.org/10.1016/S0002-9440(10)63491-8 PMID: 14507641
56. Kitchell BE, Fan TM, Kordick D, Breitschwerdt EB, Wollenberg G, Lichtensteiger CA. Peliosis hepatis in a dog infected with Bartonella henselae. J Am Vet Med Assoc. 2000 Feb; 216(4):519–23. https://doi.org/10.2460/javma.2000.216.519 PMID: 10687006
57. Breitschwerdt EB, Maggi RG, Varanat M, Linder KE, Weinberg G. Isolation of Bartonella vinsonii subsp. berkhoffii genotype II from a boy with epithelioid hemangioendothelioma and a dog with hemangiopericytoma. J Clin Microbiol. 2009 Jun; 47(6):1957–60. https://doi.org/10.1128/JCM.00069-09 PMID: 19369441
58. Mascarelli PE, Iredell JR, Maggi RG, Weinberg G, Breitschwerdt EB. Bartonella species bacteremia in two patients with epithelioid hemangioendothelioma. J Clin Microbiol. 2011 Nov 1; 49(11):4006–12. https://doi.org/10.1128/JCM.05527-11 PMID: 21918021
59. Sykes JE, Westropp JL, Kasten RW, Chomel BB. Association between Bartonella species infection and disease in pet cats as determined using serology and culture. J Feline Med Surg. 2010;
60. Brenner EC, Chomel BB, Singhasivanon O-U, Namekata DY, Kasten RW, Kass PH, et al. Bartonella infection in urban and rural dogs from the tropics: Brazil, Colombia, Sri Lanka and Vietnam. Epidemiol Infect. 2012; 141(1):1–8.
61. Cherry N, Diniz P, Maggi R, Hummel J, Hardie E, Behrend E, et al. Isolation or Molecular Detection of Bartonella henselae and Bartonella vinsonii subsp. berkhoffii from Dogs with Idiopathic Cavitary Effusions. J Vet Intern Med. 2009; 23(1):186–9. https://doi.org/10.1111/j.1939-1676.2008.0246.x PMID: 19175739
62. Perez C, Maggi RG, Diniz PPVP, Breitschwerdt EB. Molecular and serological diagnosis of Bartonella infection in 61 dogs from the United States. J Vet Intern Med. 2011; 25(4):805–10. https://doi.org/10.1111/j.1939-1676.2011.0736.x PMID: 21615498

requested document 2 - dual publication
Click here to access/download;Manuscript;Lashnits et al. 2021.pdf

pathogens

MDPI

Article

Comparison of Serological and Molecular Assays for *Bartonella* Species in Dogs with Hemangiosarcoma

Erin Lashnits [1,2], Pradeep Neupane [2], Julie M. Bradley [2], Toni Richardson [2], Ricardo G. Maggi [2,3] and Edward B. Breitschwerdt [2,3,*]

[1] Department of Medical Sciences, School of Veterinary Medicine, University of Wisconsin, Madison, WI 53713, USA; lashnits@wisc.edu
[2] Intracellular Pathogens Research Laboratory, Comparative Medicine Institute, College of Veterinary Medicine, North Carolina State University, Raleigh, NC 27606, USA; pneupan@ncsu.edu (P.N.); jmbradl3@ncsu.edu (J.M.B.); tgrichar@ncsu.edu (T.R.); rgmaggi@ncsu.edu (R.G.M.)
[3] Department of Clinical Sciences, College of Veterinary Medicine, North Carolina State University, Raleigh, NC 27606, USA
* Correspondence: ebbreits@ncsu.edu

Abstract: Currently, a gold standard diagnostic test for *Bartonella* infection in dogs is lacking. This represents a critical limitation for the development and evaluation of new diagnostic tests, as well as for the diagnosis of, and research on, bartonellosis in dogs. This retrospective observational study aims to compare the results of commonly performed and newly-reported *Bartonella* spp. diagnostic tests in banked clinical specimens from 90 dogs with hemangiosarcoma (HSA) using composite reference standard (CRS) and random effects latent class analysis (RE-LCA) techniques. Samples from each dog were tested using six serological or molecular diagnostic assays, including indirect fluorescent antibody (IFA) and Western blot (WB) for the detection of antibodies in serum, and qPCR and droplet digital PCR (ddPCR) in blood and fresh frozen tissue biopsy samples (mainly splenic HSA tumors and histopathologically normal spleen or skin/adipose tissue). *Bartonella* infection prevalence was estimated to be 78% based on the CRS (parallel testing with all six assays), and 64% based on the RE-LCA model. The assay with the highest diagnostic accuracy was qPCR performed on fresh frozen tissue biopsy samples (sensitivity: 94% by RE-LCA and 80% by CRS; specificity: 100%). When comparing newly-reported to traditional *Bartonella* diagnostic assays, ddPCR was more sensitive for the detection of *Bartonella* DNA than qPCR when testing blood samples (36% vs. 0%, $p < 0.0001$). Dogs that were positive on serological assays alone with negative molecular assays were highly unlikely (<3%) to be classified as infected by the RE-LCA model. These data indicate that *Bartonella* spp. DNA can be PCR amplified from fresh frozen tissues from a majority of dogs with HSA using both qPCR and ddPCR, supporting the use of these methods for future controlled studies comparing the prevalence of *Bartonella* spp. DNA in the tissue of dogs with HSA to that of unaffected controls.

Keywords: diagnostic testing; ddPCR; PCR; serology; sensitivity; specificity; tissue biopsy; latent class analysis

check for updates

Citation: Lashnits, E.; Neupane, P.; Bradley, J.M.; Richardson, T.; Maggi, R.G.; Breitschwerdt, E.B. Comparison of Serological and Molecular Assays for *Bartonella* Species in Dogs with Hemangiosarcoma. *Pathogens* **2021**, *10*, 794. https://doi.org/10.3390/pathogens10070794

Academic Editor: Lawrence S. Young

Received: 21 May 2021
Accepted: 18 June 2021
Published: 23 June 2021

Publisher's Note: MDPI stays neutral with regard to jurisdictional claims in published maps and institutional affiliations.

Copyright: © 2021 by the authors. Licensee MDPI, Basel, Switzerland. This article is an open access article distributed under the terms and conditions of the Creative Commons Attribution (CC BY) license (https://creativecommons.org/licenses/by/4.0/).

1. Introduction

Documentation of zoonotic bartonelloses in humans in the 1990s predated the identification of the first case of bartonellosis in a dog by only a few years [1–3]. The ongoing discovery of new *Bartonella* spp., seventeen of which have been associated with disease in dogs or humans, continues to present challenges to diagnosticians, clinicians, and patients in both human and veterinary medicine [4–7]. In human cases of zoonotic bartonellosis, diagnosis has historically relied on serology, culture from blood or tissue, and visualization of bacteria with silver stains in lymph node biopsies from patients with suspected Cat Scratch Disease (CSD) [8]. Initially developed to assess *B. henselae* seroreactivity in patients

with CSD, an indirect fluorescent antibody (IFA) assay subsequently became the reference standard for the diagnosis of bartonellosis (the best method available), and the test to which newly developed assays were compared [9,10]. Advances in serological methods and the advent of molecular-based assays have facilitated the diagnosis of bartonellosis in some clinically suspected cases; however, there remains uncertainty regarding the diagnostic accuracy of currently available tests for *Bartonella* spp. infection, particularly in patients with nonspecific, chronic symptoms, and in patients with what have been historically considered atypical manifestations of CSD [4,11,12].

Because of the fastidious growth conditions required by *Bartonella* spp., the sensitivity of culture is estimated to be only 20–30%, even with specialized culture procedures and media (5% CO_2, 35–37 °C, various specialized media), but varies substantially depending on the *Bartonella* spp., clinical presentation, tissue used for culture, and antibiotic history of the patient, among other variables [4,13–15]. PCR amplification of *Bartonella* spp. DNA from blood or tissue specimens, thought to improve diagnostic sensitivity, is now routinely performed by many commercial diagnostic laboratories. Clinically, PCR sensitivity depends not only on laboratory factors like the choice of PCR primers and *Bartonella* gene targets, the equipment and methodological considerations, but also on the clinical presentation (illness duration and other factors) and sample type (blood, other body fluid, tissue biopsy, etc.) [15–17]. With so many factors influencing the accuracy of PCR, a broad range of PCR sensitivity has been reported (33–92%), even when testing solely patients with prototypical diseases caused by *Bartonella* spp. infection, such as endocarditis and CSD [14,18]. A true gold standard test is defined by a sensitivity and specificity of 100%, so the current human reference standard of serology is far from that. Studies investigating the sensitivity and specificity of serology in *Bartonella* spp. infection have mainly focused on patients with acute CSD or culture-negative endocarditis (presumably a more chronic infection with the eventual localization of bacteria to the heart valve). Similar to reports on PCR, these studies of *Bartonella* spp. serology report highly variable sensitivity (20–90%), though specificity has been better (93–98%) [4,11,19–23]. The accuracy of serological testing in chronic bartonellosis or in manifestations other than acute CSD or endocarditis has not been rigorously assessed, though serology is thought to have poor sensitivity in these "atypical" cases based on observations that people with documented *Bartonella* spp. DNA in their bloodstream or tissues are often seronegative [24–28]. Conceptually, serology also may have poor sensitivity due to the potential for antigenic switching and immune evasion, and poor specificity for active infection since seroreactivity may persist after exposure, despite effective immunological or antibiotic elimination of the infection [18,20,29]. Indeed, finding false negative serology in actively infected dogs can be common with other infectious and vector borne diseases, particularly those with an intracellular lifestyle such as brucellosis, leishmaniasis, or fungal diseases [30–35]. Lack of specificity of IFA for active infection caused by *Bartonella* spp. is also evidenced by reports of *Bartonella* seroreactivity in apparently healthy asymptomatic people worldwide [36–38].

Challenges to definitively diagnose bartonelloses in humans are, unsurprisingly, recapitulated in companion animal veterinary medicine. Bartonellosis in dogs is found worldwide and associated with multiple acute or chronic clinical manifestations including endocarditis, pyogranulomatous inflammatory disease, vasoproliferative lesions, and nonspecific signs such as fever, lymphadenopathy, or uveitis [7]. As in humans, there are numerous species of *Bartonella* reported in dog infections, including most commonly *B. henselae*, *B. vinsonii* subsp. *berkhoffii*, and *B. koehlerae* [39,40]. For zoonotic *Bartonella* spp., accurate diagnosis of *Bartonella* infection in dogs as sentinel species is of paramount importance to informing our evolving understanding of *Bartonella* spp. transmission and the spectrum of disease manifestations attributable to this genus of bacteria [41,42]. There is currently no accepted gold standard test–or test free from error–for the diagnosis of bartonellosis in dogs, either for clinical cases or for use in epidemiologic or clinical research [7,43]. A limited number of studies have investigated the diagnostic accuracy of commonly used *Bartonella* assays in naturally infected dogs [44–46]. PCR sensitivity when

testing blood, compared to tissue as the specimen source, or when compared to enrichment blood or tissue culture using the *Bartonella* α-proteobacteria growth medium (BAPGM) platform, appears particularly poor, with estimates ranging from 0% up to approximately 50% [46–49]. Serology has been similarly problematic, with sensitivity estimates ranging from less than 40% up to approximately 60% in naturally infected dogs, despite sensitivities of 100% in experimentally infected dogs [44,46,50,51]. Additionally, multiple studies have suggested a poor correlation between the species/strain IFA seroconversion and *Bartonella* species/strain based on PCR in naturally infected dogs, despite accurate species/strain seroconversion in experimentally infected dogs [44–47,49,50]. In the absence of a gold standard diagnostic test, estimates of specificity are difficult to obtain in naturally infected dogs. However IFA specificity has been estimated at over 85% when compared to a panel of previously performed diagnostic tests [44]. Additionally, among a large sample of over 15,000 North American dogs suspected of vector-borne disease, only 4% were *Bartonella* spp. seroreactive, suggesting that seroreactivity against *Bartonella* spp. is fairly uncommon even in dogs with potential for vector exposure [39]. Despite the commercial availability of *Bartonella* serology and PCR through many veterinary diagnostic laboratories, diagnostic accuracy metrics are not well established, mainly due to limitations shared with human diagnostic testing modalities–specifically the use of imperfect reference standards for "gold standard" comparisons [44,52].

Because of these historical and current diagnostic limitations, recent efforts have been directed at ongoing improvements in serological and molecular assays that are applicable to both companion animals and human patients [53–55]. Though available commercially for over 20 years, validation of interpretation criteria for WB serodiagnosis of bartonelloses in dogs was only recently published [56,57]. Using a combination of naturally and experimentally infected dogs, sensitivity and specificity of WB was estimated at 53% and 96% respectively [56]. In addition, to improve upon current molecular diagnostics, droplet digital PCR (ddPCR)—a technology originally developed to precisely estimate DNA copy number–has been adapted to the detection of rare or low abundance pathogen DNA in a background of abundant host DNA, and applied to the diagnosis of bartonellosis. The analytical and diagnostic validation of ddPCR for detection of *Bartonella* spp. DNA in human blood samples was recently published, with an estimated sensitivity between 50–75% depending on the reference standard, and estimated specificity of 99% [28,58]. This method has not previously been reported for use in companion animal species.

There has been extensive research into statistical methods to evaluate diagnostic accuracy of imperfect tests when no gold standard reference test exists [59–61]. Historically, a composite reference standard (CRS) could be defined using a combination of imperfect tests as a reference, but this method relies heavily on the utility of each test and the correct classification of a subject [62,63]. Another approach, known as latent class analysis (LCA), uses probabilistic modeling to classify patients into disease states (the so-called latent variable, here infected or uninfected) based on the observed results of their imperfect diagnostic tests. In LCA, the model-based classification is then used to estimate diagnostic accuracy parameters (sensitivity and specificity), even in the absence of a gold standard.

In a previous published study from our research group, we obtained a specimen set from dogs histopathologically diagnosed with hemangiosarcoma (HSA), and determined that the prevalence of *Bartonella* spp. DNA (indicative of *Bartonella* infection in these dogs) was remarkably high (73%) [47]. The availability of multiple specimen types (serum, blood, and fresh frozen tissue biopsies) from each dog, as well as the overall high proportion of dogs with evidence of *Bartonella* spp. infection, made this a uniquely useful sample set in which to compare results from two newly developed assays (WB, ddPCR) to multiple commonly performed and commercially available assays (IFA, qPCR), in both blood and tissue samples. The objective of this study was to estimate clinical sensitivity and specificity of these commonly performed and newly-reported diagnostic assays for detection of *Bartonella* spp. across multiple sample types. Our hypothesis was that WB and ddPCR

would be more sensitive for detection of *Bartonella* antibodies or DNA in blood and tissue samples from naturally infected dogs, when compared to IFA and qPCR assays.

2. Results

There were 90 dogs included in this study, with a median age of 10 years (range 4–20 years); 51% of the dogs were female, and the most common breeds were mixed breed (24), Labrador retriever (17), and Golden retriever (11), with other breeds each representing ≤5% of the study group. When results from all six assays (IFA, WB, qPCR on blood, qPCR on tissue, ddPCR on blood, and ddPCR on tissue) were evaluated in parallel, *Bartonella* DNA and/or antibodies were found in 70 of the 90 dogs (78%). There were 59 dogs (66%) that had a positive result on any two or more assays. The serology and PCR results for each of the six assays are shown in Table 1. The RE-LCA and CRS estimated sensitivity and specificity for each assay are shown in Table 1. Though not clinically practical, to maximize the sensitivity of detection of *Bartonella* spp. an inclusive CRS was defined for sensitivity calculations using the combined results of all six assays in parallel: a dog was considered positive on the inclusive reference standard if it had a positive result on any one or more of these six assays, and considered negative it was negative on all six assays. Since specificity could not be calculated with this inclusive CRS, a separate CRS was defined for calculation of specificity of each test: for the specific CRS, a dog was considered positive if it had a positive result on any two or more of the six assay, and considered negative it was negative on all six assays or positive on only one assay. Based on RE-LCA, 64% of dogs were classified as infected. The conditional probability of a dog being classified as *Bartonella* infected or not *Bartonella* infected, based on RE-LCA, for each test is shown in Figure 1.

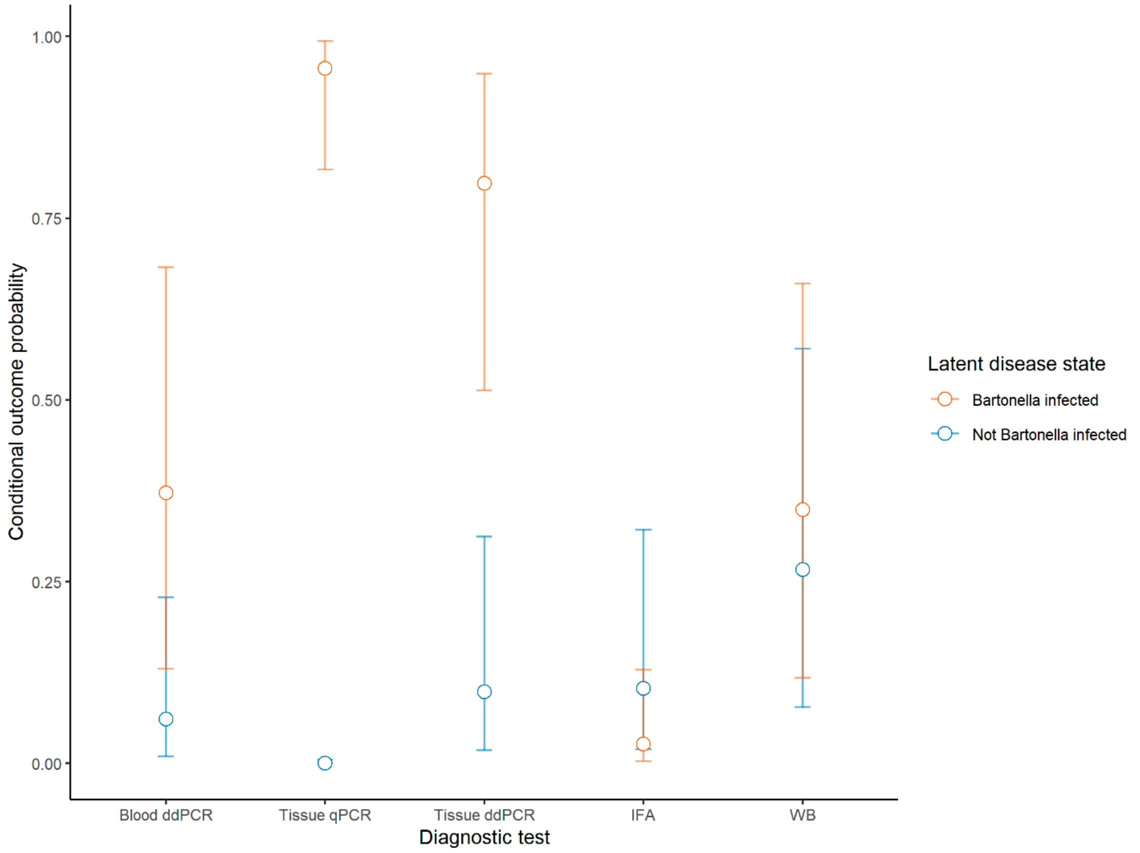

Figure 1. Conditional outcome probabilities of a positive result for class (*Bartonella* infected, in orange or Not *Bartonella* infected, in blue) for each test. The conditional outcome probability is the probability of a positive result for a subject with a random effect of zero (an "average" subject). Points show the probability estimate calculated based on RE-LCA, and bars show the 95% confidence interval for the estimate.

Table 1. PCR and serology results for each of six different *Bartonella* diagnostic assays. The table shows the number of dogs positive by each assay, sensitivity and specificity of each assay estimated from RE-LCA, sensitivity of each assay or combination of assays compared to the CRS defined for this study, and the number of dogs positive on each individual assay but negative on the remaining five assays. The inclusive reference standard was defined for this study as a combination of results from all six assays evaluated in parallel.

	Positive Dogs (n = 90)	% Sensitivity (95% CI)		% Specificity (95% CI)		Dogs Positive Solely by This Method
		RE-LCA	CRS	RE-LCA	CRS	
Individual molecular tests						
Blood qPCR	0	–	0 (0–5.2)	–	NA	0
Blood ddPCR	25	38 (25–54)	36 (26–47)	92 (80–100)	100 (89–100)	0
Tissue ddPCR	50	78 (65–98)	71 (60–81)	88 (70–100)	94 (79–98)	2
Tissue qPCR	56	94 (79–100)	80 (69–88)	100 (71–100)	87 (71–95)	4
Individual serology tests						
IFA	6	4 (0–9)	8.6 (4.0–18)	88 (72–100)	100 (89–100)	0
WB	30	36 (23–49)	43 (32–55)	72 (50–89)	84 (67–93)	5
Combinations of tests						
Any serology test	32	–	46 (35–57)	–	84 (67–93)	–
Any molecular test	64	–	91 (83–96)	–	81 (64–91)	–
Tissue qPCR + IFA (in parallel)	60	–	86 (76–92)	–	87 (71–95)	–
Any of the six assays (CRS sensitivity)	70	–	*reference*	–	65 (47–79)	–
2 or more of the six assays (CRS specificity)	59	–	84 (74–91)	–	*reference*	–

In this group of dogs with HSA, the single assay with the highest sensitivity was qPCR performed on fresh frozen tissue biopsy samples (94% [95% CI 79–100%]). This assay also had the highest estimate specificity (100% [95% CI 71–100%]). For each individual assay, Table 1 also reports the number of dogs that were only positive on the respective assay, but negative by the remaining five assays. The Tissue qPCR had the highest sensitivity with the lowest false positive rate. While tissue ddPCR, blood ddPCR, and IFA had similar false positive rates, they differed widely in sensitivity. The observed and expected frequencies of each combination of assay results, along with the probability that a dog with each combination of results would be classified as infected by the RE-LCA model, are shown in Table 2.

When comparing newly-reported to traditional *Bartonella* diagnostic assays, ddPCR was more sensitive than qPCR for detection of *Bartonella* DNA when testing blood samples (36% vs. 0%, $p < 0.0001$), though it was not when testing tissue samples (71% vs. 80%, $p = 0.324$). Based on the kappa value, there was moderate agreement between qPCR and ddPCR when both assays were performed on tissue biopsy samples (kappa = 0.59, Table 3). Tissues from 12 dogs were qPCR positive but ddPCR negative (i.e., 21% of the qPCR positive tissues were ddPCR negative), compared to tissues from only six dogs that were ddPCR positive but qPCR negative (i.e., 12% of the ddPCR positive tissues were qPCR negative). Based on the RE-LCA model, a dog with a positive result on tissue qPCR had a >99% probability of being classified as infected, regardless of the other assay results (positive or negative). Based on the kappa value, there was no to slight agreement between WB and IFA when both assays were performed on serum samples (kappa = 0.13, Table 4). The combined results from the two most commonly used assays taken in parallel (serum IFA and blood qPCR) had 9% sensitivity when compared to the reference standard.

Table 2. Results of RE-LCA model. For each combination of assay results observed in the dataset, the number of dogs is shown. Observed indicates the number of dogs with the specified pattern counted in the dataset. Expected indicates the number dogs with the specified pattern predicted by the RE-LCA model. The probability of being classified as infected or not infected, as predicted by the RE-LCA model, for a dog with each specified pattern, is also shown. For each assay result, a 1 indicates a positive result and a 0 indicates a negative result.

	**Assay Result**					**Number of Dogs**		**Probability of Classification**	
Result summary	Blood ddPCR	Tissue qPCR	Tissue ddPCR	IFA	WB	Observed	Expected	Infected	Not infected
Blood ddPCR + Tissue qPCR + Tissue ddPCR only positive	1	1	1	0	0	11	9.7	1.0	0.0
All positive except IFA	1	1	1	0	1	6	7.3	1.0	0.0
Tissue qPCR + Tissue ddPCR only positive	0	1	1	0	0	16	16.4	1.0	0.0
Tissue qPCR + Tissue ddPCR + WB only positive	0	1	1	0	1	9	8.8	1.0	0.0
Blood ddPCR + Tissue qPCR only positive	1	1	0	0	0	3	2.4	1.0	0.0
Blood ddPCR + Tissue qPCR + WB only positive	1	1	0	0	1	1	1.3	0.999	0.001
Tissue qPCR + Tissue ddPCR + IFA only positive	0	1	1	1	0	1	0.5	0.999	0.001
Tissue qPCR only positive	0	1	0	0	0	4	5.9	0.999	0.001
All positive except blood ddPCR	0	1	1	1	1	1	0.4	0.998	0.002
Tissue qPCR + WB only positive	0	1	0	0	1	4	2.2	0.998	0.002
Blood ddPCR + Tissue ddPCR only positive	1	0	1	0	0	2	0.6	0.720	0.280
Tissue ddPCR only positive	0	0	1	0	0	2	2.9	0.410	0.590
Blood ddPCR + WB only positive	1	0	0	0	1	1	0.6	0.099	0.901
Tissue ddPCR + IFA only positive	0	0	1	1	0	1	0.3	0.059	0.941
Tissue ddPCR + IFA + WB only positive	0	0	1	1	1	1	0.2	0.044	0.956
All assays negative	0	0	0	0	0	20	17.4	0.037	0.963
WB only positive	0	0	0	0	1	5	5.9	0.027	0.973
Blood ddPCR + IFA + WB only positive	1	0	0	1	1	1	0.1	0.010	0.990
IFA + WB only positive	0	0	0	1	1	1	0.9	0.003	0.997

Table 3. Comparison between qPCR and ddPCR when testing tissue biopsy samples from dogs with HSA. Number and percentage of all dogs (n = 90) with positive or negative result for each assay shown.

		Tissue qPCR Result	
		Positive	Negative
**Tissue ddPCR result**	Positive	44 (49%)	6 (6.7%)
	Negative	12 (13%)	28 (31%)

Table 4. Comparison between WB and IFA when testing serum samples from dogs with HSA. Number and percentage of all dogs (n = 90) with positive or negative result for each assay shown.

		IFA Result	
		Positive	Negative
**WB result**	Positive	4 (4.4%)	26 (29%)
	Negative	2 (2.2%)	58 (64%)

When comparing combined molecular results with combined serologic results, molecular testing was more sensitive for detection of *Bartonella* spp. (molecular testing 91% sensitivity, serology 46% sensitivity, $p < 0.0001$). A dog was considered molecular assay positive if *Bartonella* spp. DNA was PCR amplified from one or more blood or tissue sample by qPCR, ddPCR, or both. A dog was considered serologically positive if it was IFA seroreactive and/or WB positive. When comparing overall *Bartonella* spp. molecular results with serologic results, there was slight to no agreement (kappa = 0.13, Table 5). Of 64 dogs with *Bartonella* DNA amplified from blood or tissue, only 41% (26 dogs) were seroreactive. Conversely, 19% of the 32 seroreactive dogs did not have DNA amplified from blood or tissue. Based on the RE-LCA model, dogs that were positive on serology alone (WB only, or WB and IFA) with negative molecular assays were highly unlikely (<3%) to be classified as infected.

Table 5. Comparison between Bartonella serological and molecular assay results for 90 dogs diagnosed with HSA. Molecular assays included qPCR and ddPCR on blood and tissue; serologic assays included IFA and WB. Number and percentage of all dogs (n = 90) with positive or negative result for each combination of assays shown.

		Molecular Result	
		Positive	Negative
**Serology result**	Positive	26 (29%)	6 (6.7%)
	Negative	38 (42%)	20 (22%)

When comparing combined molecular assay results between blood and tissue samples, tissue molecular testing was more sensitive for the detection of *Bartonella* spp. DNA (blood molecular sensitivity 36%, tissue molecular sensitivity 89%, $p < 0.0001$). A dog was considered tissue molecular test positive if *Bartonella* spp. DNA was PCR amplified from one or more tissue biopsy samples using qPCR, ddPCR, or both (each dog had two tissue biopsy samples tested). A dog was considered blood molecular test positive if *Bartonella* spp. DNA was PCR amplified from its blood sample using qPCR, ddPCR, or both. Based on the kappa value, there was fair agreement between the molecular assay results when performed on blood samples compared to tissue samples (kappa = 0.22, Table 6). Of 62 dogs with *Bartonella* spp. DNA PCR amplified from tissue samples, only 37% (23 dogs) also had *Bartonella* DNA PCR amplified from blood samples. The combined result from three assays taken in parallel (WB, tissue qPCR, and tissue ddPCR) had 100% sensitivity when compared to the inclusive reference standard.

Table 6. Comparison between molecular testing on blood and tissue. A positive tissue molecular test was defined as a dog that had Bartonella DNA amplified from one or more tissue samples using qPCR, ddPCR, or both. A positive blood molecular test was defined as a dog that had Bartonella DNA amplified from one or more blood samples using qPCR, ddPCR, or both. In this sample of 90 dogs with HSA, all blood qPCR results were negative.

		**Tissue Molecular Result**	
		Positive	Negative
**Blood molecular result**	Positive	23	2
	Negative	39	26

Bartonella species could be identified with Sanger sequencing of PCR products from qPCR. Since no dog had *Bartonella* spp. DNA amplified from a blood sample by qPCR, *Bartonella* spp. identity could only be determined from tissue biopsy samples. In these tissue biopsies from dogs with HSA, *B. henselae* DNA was most often amplified. Homologies ranged from 99.3% to 100% (of the 138 bp analyzed) with *B. henselae* CAL1 and SA2 (Genbank accessions AF369527 and AF369529, respectively). Of the 56 *Bartonella* qPCR positive tissues, 52 (93%) contained *B. henselae* DNA, including 1 dog that had both *B. henselae* and *B. koehlerae* DNA PCR amplified from tissue. One dog had only *B. koehlerae* DNA PCR amplified from tissue (140/140 bp, 100% homology with Genbank accession AF312490). In two dogs, the *Bartonella* species could not be determined for the DNA sequences amplified; for these two samples, homology ranged from 95% to 98% with either *B. henselae* CAL1 or *B. henselae* SA2.

Two biopsy samples were tested with each molecular assay. There were 12 dogs that had *Bartonella* spp. DNA ddPCR amplified from both biopsy samples and 38 that had *Bartonella* spp. DNA ddPCR amplified from one of the two biopsy samples. There were 15 dogs that had *Bartonella* spp. DNA qPCR amplified from both biopsy samples (of these, 9 had the same species and strain, based on Sanger sequencing of PCR products), and 41 that had *Bartonella* spp. DNA qPCR amplified from one of the two biopsy samples.

3. Discussion

In this study estimating sensitivity and specificity of *Bartonella* spp. diagnostic assays, 62% of dogs with histopathologically confirmed HSA and multiple *Bartonella* spp. assays performed were classified as infected with >99% probability based on latent class analysis. *Bartonella* qPCR performed on fresh frozen tissue biopsy samples had the highest estimated sensitivity and specificity (94% and 100% respectively) of any single assay. A dog with a positive result on tissue qPCR had a >99% probability of being classified as infected, regardless of the other assay results (positive or negative).

When comparing newly-reported to traditional *Bartonella* diagnostic assays, ddPCR was not more sensitive for detection of *Bartonella* DNA than qPCR when testing tissue samples (78% vs. 94% sensitivity). Though 70 dogs in this study had molecular and/or serological evidence of exposure to or infection with a *Bartonella* spp. when tested with all six different assays, not a single dog had *Bartonella* spp. DNA amplified from blood by qPCR, and only 8% were seroreactive by IFA. Based on RE-LCA, four of the six IFA positive dogs had >94% probability of being classified as not infected, demonstrating that while IFA is more specific than sensitive, the specificity is unlikely to be perfect. In contrast, half of the dogs positive by WB were classified as not infected–these included mainly dogs that were positive by WB alone or by WB and IFA (but negative on all molecular assays).

While qPCR did not amplify *Bartonella* DNA from any dog's blood sample, ddPCR proved a more sensitive DNA detection method from the blood. However, even ddPCR performed on blood samples had an estimated sensitivity of only 38%. There were 39 dogs in this study that had *Bartonella* DNA amplified from their tissue samples, but both ddPCR and qPCR testing of blood were negative. Previous studies in dogs and humans have shown very good analytical accuracy and high specificity of well-designed PCR assays [24,46,64].

Despite this, the discrepancy between PCR amplification of DNA from blood and tissue samples is also commonly reported in recent studies [47–49,51]. Recent examples include an experimental study in which bacteremia was never confirmed in dogs experimentally infected with *Bartonella* spp., despite viable *Bartonella* spp. being isolated from tissues (bone marrow and lung) collected at the time of post-mortem examination [51]. In another clinically relevant report, a dog had *B. henselae* DNA PCR amplified from biopsies of vasculitis lesions and normal skin, but *Bartonella* DNA was not PCR amplified, even after BAPGM enrichment culture, from multiple sequential blood samples [65].

Proposed explanations for lower sensitivity of both qPCR and ddPCR in blood compared to tissue include both the variable nature of *Bartonella* spp. bacteremia, as well as the stochastic and dilutional phenomena associated with small quantities of *Bartonella* spp. DNA in patient blood samples. DdPCR was specifically designed to detect low-copy-number DNA on a background of abundant host DNA, and there were 25 dogs in this study that had qPCR negative but ddPCR positive blood samples. This supports the theory that small quantities of *Bartonella* spp. DNA in blood samples limits the clinical sensitivity of qPCR; ddPCR appears to improve upon this limitation. Testing multiple blood samples per dog (similar to the "triple draw" technique recommended for blood sampling for *Bartonella* PCR/BAPGM in humans) [66] might further increase the diagnostic sensitivity of both qPCR and ddPCR performed on blood samples, though this has not been specifically evaluated previously in dogs. In addition, BAPGM enrichment culture on blood samples might improve sensitivity [45,46], but the blood samples provided for this study were not collected or processed aseptically so enrichment culture was not possible. The sampling protocol used in this study may be another explanation for the higher sensitivity of molecular testing performed on tissues compared to blood samples. Because of the originally determined sampling protocol for the CCOGC, dogs had two biopsy samples obtained and submitted. Considering tissue results using two samples likely increased the sensitivity of this assay compared to using only one sample for molecular testing of blood. Indeed, there were only nine dogs for which the same *Bartonella* spp. DNA was amplified by qPCR from both tissue biopsy samples.

Adding to the difficulty interpreting the clinical significance of *Bartonella* bacteremia, as indicated by PCR positive blood samples, *Bartonella* spp. bacteremia can also be fairly common in apparently healthy dogs. One study of healthy volunteer dogs in North Carolina found that approximately 10–20% had evidence of *Bartonella* spp. bacteremia based on BAPGM enrichment blood culture/PCR [67]. The reasons that *Bartonella* DNA is not cultured or PCR amplified from blood samples from dogs with *Bartonella* DNA present in tissue could include intermittent bacteremia, rapid clearance of bacteremia by the dogs' immune system, a transient bacteremic phase of infection prior to a chronic tissue phase, or lower concentrations of bacterial DNA in blood compared to tissue. Regardless of the mechanism(s), *Bartonella* PCR performed on a single blood sample does not effectively predict whether a dog has *Bartonella* spp. DNA in its tissues.

It is important to emphasize that fresh frozen rather than formalin fixed paraffin embedded (FFPE) tissues were used for the molecular assays performed in this study. Formalin fixation decreases PCR sensitivity by making DNA extraction more difficult, mainly because of chemical modification, as well as DNA trapping and potentially DNA fragmentation [68]. Though head-to-head comparisons of *Bartonella* spp. ddPCR on fresh frozen compared to FFPE tissues have not been reported, based both on the mechanism by which formalin fixation decreases qPCR sensitivity, as well as studies of other infectious diseases, it is reasonable to assume that ddPCR sensitivity is also decreased when performed on FFPE tissue samples compared to fresh frozen samples [69]. Thus, it is of critical importance that clinicians collect and store frozen tissue at the time of biopsy, surgery, or autopsy if PCR-based molecular diagnostics are to be performed.

While we were fortunate to have a set of samples for this study that included two biopsy samples of tissue as well as blood and serum, obtaining tissues is an invasive procedure that carries risks for complications and is generally accompanied by substantial

sampling expense. These practical constraints may limit the widespread clinical utility of tissue biopsies for routine *Bartonella* testing. However, it is notable that many of the tissues included in this study were skin biopsies from normal-appearing skin in dogs with pathology in distant organs. Skin biopsies can in fact often be obtained at substantially less risk and expense than biopsies of other potentially infected organs (spleen, liver, heart). The distribution of *Bartonella* in tissues is not likely uniform, but given the ability for *Bartonella* to induce pathology systemically via secreted factors such as BafA, and to infect vascular endothelial cells which are distributed throughout the body, it is possible that *Bartonella* infection in the skin may ultimately be shown to correlate with systemic illness [70,71]. However the importance and variability of the localization of *Bartonella* infection in any given organ is poorly characterized at this time [70].

In regard to *Bartonella* serology, when both WB and IFA results were considered in parallel *Bartonella* spp. serology was positive in less than half (36%) of HSA dogs that had *Bartonella* spp. DNA amplified from blood or tissue. Based on the RE-LCA model, dogs that were positive on serology alone (WB only, or WB + IFA) with negative molecular assays were highly unlikely (<3%) to be classified as infected. Previous studies documenting poor agreement between *Bartonella* spp. IFA seroreactivity and *Bartonella* spp. bacteremia based on PCR/BAPGM in dogs have been reported [44,46,72]. IFA is known to have low sensitivity and may underestimate the true seroprevalence of *Bartonella* spp. exposure in dogs [44,46,73–75]. IFA in this group of dogs with HSA had a lower sensitivity and specificity (4% and 88% respectively) than has been historically reported, with one early study estimating *Bartonella* spp. IFA sensitivity at approximately 40% when compared to documentation of active infection by BAPGM enrichment blood culture/PCR [46], and a more recent study estimating a 62% sensitivity when using an expanded IFA panel including eight *Bartonella* spp. antigens, again compared to positive BAPGM enrichment blood culture/PCR results [44]. Possible explanations for the lower IFA sensitivity reported here may be due to immune evasion or modulation at different stages of infection, since previous reports have investigated BAPGM enrichment blood culture/PCR rather than tissue biopsy samples, and blood samples do not always accurately reflect the status of tissue infection. Since none of the dogs in this report had *Bartonella* spp. DNA amplified from blood samples using qPCR, we cannot evaluate the sensitivity/specificity of IFA in dogs positive only by qPCR performed on blood samples.

WB had higher sensitivity compared to IFA for detecting *B. henselae* antibodies (36% vs. 4%), but also lower specificity and a higher false positive rate. The five dogs positive solely by WB had a 97% probability of being classified as not infected based on the RE-LCA model (false positives). Because it is not possible to distinguish whether the antibodies detected by WB or IFA are indicative of active infection or previous exposure with effective clearance of bacteria, it is possible that seroreactive dogs were previously exposed, but not actively infected with a *Bartonella* spp. Conversely, for the 38 dogs that had *Bartonella* spp. DNA PCR amplified from blood and/or tissue, but were nonseroreactive, it is possible that the bacterial strategies used to evade the immune system in intracellular infection contribute to a lack of detectable antibodies by either IFA or WB.

This study was designed to be primarily descriptive, and as such has multiple limitations. Importantly, there was no control group consisting of confirmed *Bartonella*-negative dogs with which to evaluate test specificity in similar manner to the traditional method using the inclusive reference standard to estimate sensitivity, and therefore ground our RE-LCA model in observed data. Using RE-LCA precludes the need for "known" disease status, however, and allows estimation of both sensitivity and specificity without a control population. Previous studies attempting to identify populations of dogs that had never been exposed to, or infected with, *Bartonella* spp. to serve as controls have often encountered considerable difficulty. For example, in an early study attempting to develop an experimental infection model of *B. henselae* and *B. vinsonii* subsp. *berkhoffii*, the two dogs obtained from a university breeding facility were infected with *B. koehlerae* prior to initiation of the study [51]. This, and other studies showing *Bartonella* infection or exposure

in dogs expected to be unexposed/uninfected, illustrate the difficulty of finding a truly negative dog population with which to evaluate test specificity [49,67]. In an attempt to minimize false negatives, we included molecular assay results on both of two distinct tissue biopsies submitted or each dog; this likely increased the sensitivity of molecular assays performed on tissue compared to blood samples (of which only one sample per dog was tested). Future studies evaluating diagnostic accuracy metrics for *Bartonella* spp. assays should use the same number of specimens for each sample type, and consider evaluating multiple blood samples to determine the degree to which repeated sampling improves molecular assay sensitivity in this sample type. In addition, future studies could consider using Bayesian techniques to evaluate diagnostic accuracy of *Bartonella* spp. assays. LCA using a Bayesian framework has been successfully performed for decades, and allows for greater flexibility in model creation as well as the use of informed priors that could be based on this and other studies [76–78].

Another limitation of this study concerns using PCR (qPCR or ddPCR) as a diagnostic method, because demonstration of pathogen DNA in a patient sample does not necessarily mean that live/viable pathogen is present in the patient, nor that that pathogen is the cause of the patient's clinical disease [79]. For *Bartonella* spp., demonstration of live/viable bacteria is possible with culture or BAPGM-ePCR; unfortunately the samples in this study were not collected sterilely, precluding BAPGM-ePCR. Finally, because this was a retrospective study using a previously obtained set of samples from dogs with hemangiosarcoma, the results of this study cannot be applied to the diagnosis of other clinical or pathological disease presentations in dogs.

Currently, the "gold standard" of bartonellosis diagnosis (in dogs or humans) continues to rely on a combination of culture, serology, and molecular tests with poor, variable, or undetermined sensitivity, specificity, or both. In the absence of a true gold standard, it is difficult to accurately estimate clinical sensitivity and specificity of diagnostic tests. Without this information, it is impossible for clinicians to critically interpret test results, for epidemiologists to draw appropriate conclusions about population-wide trends, or for vector biologists and ecologists to clarify modes and routes of transmission. When clinical diagnostic tests are assumed to be more sensitive than they are, false negatives are not recognized and serious or fatal cases of bartonellosis may go undiagnosed [24,80]. False positives may also lead to inaccurate diagnosis and potentially inappropriate treatments, particularly with unnecessary antibiotics. Our findings emphasize the need to further investigate the sensitivity and specificity of serological and molecular diagnostic tests for *Bartonella* spp.—both those assays currently in commercial use and novel assays that are continuing to be developed–in a variety of clinical settings. These findings also illustrate the limitations and diagnostic complexity associated with determination of *Bartonella* spp. exposure and infection in clinical patients, particularly as IFA and blood qPCR–the two most common diagnostic assays used by clinicians–had poor diagnostic accuracy. As many epidemiology studies are also based solely on these assays, the lack of sensitivity of these assays illustrated in this and other studies should be considered by researchers attempting to define *Bartonella* disease manifestations, ecology, transmission, and zoonotic risks.

4. Methods

4.1. Study Design and Sample Sources

This was a retrospective, observational, descriptive study involving 90 dogs with HSA. Specimens used for this study were previously collected from eight university veterinary hospitals across the United States between May 2008 and November 2011 and were banked by the Canine Comparative Oncology and Genomics Consortium (CCOGC) based on previously published standard operating procedures. [81] These samples were used in a previous study from our laboratory to determine the proportion of dogs with HSA that had hemotropic pathogen DNA (*Babesia, Bartonella,* and hemotropic *Mycoplasma* spp.) in their blood or tissues [47]. The CCOGC provided demographic information for each dog, including age (years), breed, weight (kg), and sex and neuter status. The date and

geographic location (university veterinary hospital) of sample collection was also provided. The anatomic location and histopathological diagnosis of HSA for each dog was also provided, and confirmed as previously described [47].

There were 110 eligible dogs from the originally published study [47]; of these, 20 dogs were missing one or more sample type and were therefore excluded, resulting in complete specimen set from 90 dogs that were ultimately used to generate the results reported in this study. Three specimen types were defined for pathogen testing: whole blood, serum, and fresh frozen tissue biopsies. For tissues, two biopsy samples were tested for each dog. The majority of tissues submitted were biopsies from splenic HSA tumors (64), histopathologically normal spleen (27), and histopathologically normal skin and/or adipose tissue (29); other tissues (60) included cardiac (HSA tumor and nontumor tissue), skeletal muscle, liver (HSA tumor and nontumor tissue), lung, kidney, mammary gland, and tissues of undetermined origin.

4.2. Bartonella Detection Methods

Traditional diagnostic tests for *Bartonella* spp., including IFA and qPCR on blood and tissue, were performed as part of the previous study [47]. For IFA testing, *Bartonella* antibodies were determined using three *Bartonella* spp. grown in cell culture (*Bartonella henselae*, *Bartonella vinsonii* subsp. *berkhoffii*, and *Bartonella koehlerae*) as antigens and following standard immunofluorescent antibody assay (IFA) techniques [47]. Sera were first screened at dilutions of 1:16 to 1:64. All sera that are reactive at 1:64 were further tested with two-fold dilutions to 1:8192. A dog was considered IFA seroreactive if it was reactive at ≥1:64 against any one or more of the three *Bartonella* spp. antigens. For qPCR, each sample was screened for the presence of *Bartonella* spp. DNA using primers targeting the 16S-23S intragenic transcribed spacer (ITS) region of *Bartonella* spp., using 5 µL of template DNA as previously described [47,82]. Validation of positive qPCR results was performed by Sanger sequencing of amplicons followed by chromatogram evaluation and sequence alignment using Contig-Express and Align X software (Vector NTI Suite 10.1, Invitrogen Corp, Carlsbad, CA, USA). For bacterial species identification, DNA sequences were analyzed for nucleotide sequence homology at NCBI nucleotide database using BLAST version 2.0. A dog was considered *Bartonella* spp. qPCR positive on tissue if one or more of the biopsy samples was qPCR positive (biopsy samples run in parallel). Stringent processing methods were used to avoid DNA carryover during tissue processing [47,58,83].

For the current study, each DNA sample was screened for the presence of *Bartonella* spp. DNA using ddPCR with the same primers and probes employed for qPCR and minor modifications [58]. The procedure was amended and validated for dogs as described for human testing [58]. DNA extractions from *B. henselae*, *B. quintana*, *B. koehlerae* or *B. vinsonii subsp. berkhoffii* genotype II spiked into negative control dog blood samples (at a concentration of 0.5 bacteria/µL) were used to set threshold values and determine the limit of detection. ddPCR amplification of the dog housekeeping gene was also performed using primers and probes employed for qPCR, as a reference target to facilitate quantification. Due to instrument design limitations, digital PCR droplets are not able to be sequenced. WB was performed as previously described using *B. henselae* SA2 whole-cell protein lysates and goat antidog whole IgG [56]. As previously validated, a dog was considered WB positive if seroreactive to any two or more *B. henselae* immunodominant proteins with a molecular size of 13, 17, 29, 50, 56, and 150 kDa (allowing for a ±1 kDa margin of error in interpretation) [56].

4.3. Study Size and Statistical Methods

Data analysis was performed using R 4.0.2 (https://www.R-project.org/, accessed on 25 June 2020). The percentage of dogs positive for each individual assay, as well as different combinations of assays, were calculated. Diagnostic test results were presented in contingency tables. In the absence of an accepted gold standard for diagnosis of bartonellosis in

dogs, two methods were used to estimate diagnostic test parameters: latent class analysis with random effects (RE-LCA) and composite reference standard (CRS) techniques.

In LCA models, the true disease status is unobserved (the so-called latent variable) and diagnostic parameters are estimated based on this latent state. Using this method, a single gold standard diagnostic test is not needed, but rather each test is assumed to be imperfect and the probability of a patient being classified into each disease state is determined based on the observed combination of diagnostic test results. Classical LCA is based on the assumption of conditional independence; that is, test results for each subject are assumed to be independent, conditional on the latent state. This assumption is not necessarily met for the patients in this study: conditional independence of IFA and WB cannot be assumed, since both are based on detection of antibodies, and conditional independence of ddPCR and qPCR also cannot be assumed, since both use the same gene targets. There may also be heterogeneity between patients that is not dependent on their classification (infected or noninfected), for example based on infection severity, location, or duration. Therefore LCA with random effects was performed to account for heterogeneity among patients within each classification, with a normally distributed random effect added for each subject. RE-LCAwas done using the "randomLCA" package in R. [84] The five assays described above were used as observed indicators. Random effects LCA models using equal loading and probit scaling of the random effect were created. To investigate the possibility that *Bartonella* infection is not a binary outcome (infected vs. not infected), models with one, two, and three latent classes were compared. The number of quadrature points was left at default (quadpoints = 21). Model selection was based on BIC [84]. The minimum BIC was obtained using the model with two latent classes, and this model was therefore selected. Based on this model, classification as "*Bartonella* infected" or "Not *Bartonella* infected" was determined with membership probabilities for each class computed from the estimated model parameters. Sensitivity and specificity (and their 95% confidence intervals) were calculated based on marginal outcome probabilities.

The use of a composite reference standard (CRS) is an alternative method by which to assess test accuracy using a combination of imperfect tests [62,63,85,86]. For this study, an inclusive CRS was defined for sensitivity calculations in order to maximize the sensitivity of detection of *Bartonella* spp. infection/exposure and decrease the likelihood of false negatives. The inclusive CRS was defined using the combined results of all six assays in parallel: a dog was considered positive on the reference standard if it had a positive result on any one or more of these six assays, and considered negative it was negative on all six assays. Since specificity could not be calculated with this inclusive CRS, a separate CRS was defined for calculation of specificity of each test: for the specific CRS, a dog was considered positive if it had a positive result on any two or more of the six assay, and considered negative it was negative on all six assays or positive on only one assay. For the purpose of this study, a dog was considered to have evidence of *Bartonella* spp. infection if it had *Bartonella* spp. DNA PCR amplified on one or more assay, and considered to have *Bartonella* spp. exposure if it was IFA seroreactive or WB positive. With 90 dogs, we were able to estimate 95% confidence intervals (CIs) for the sensitivity of each test within a margin of ±approximately 11% These CIs for the sensitivity of each test were calculated using the Wilson score interval. *Bartonella* spp. positive proportions were compared using chi-squared tests with continuity correction. Statistical significance was considered when $p < 0.05$. To determine agreement between tests, the kappa statistic was calculated [87].

Author Contributions: Conceptualization, E.L., P.N., R.G.M., E.B.B.; methodology, E.L., P.N., R.G.M., E.B.B.; formal analysis, E.L.; investigation, P.N., J.M.B., T.R., R.G.M.; resources, E.B.B.; data curation, E.L., P.N., R.G.M.; writing—original draft preparation, E.L., E.B.B.; writing—review and editing, E.L., P.N., E.B.B.; visualization, E.L.; supervision, E.L., R.G.M., E.B.B.; project administration, E.L., J.M.B., E.B.B.; funding acquisition, R.G.M., E.B.B. All authors have read and agreed to the published version of the manuscript.

Funding: This research was supported by the American Kennel Club Canine Health Foundation (CHF Grant No. 2550, EBB and MB, Feb 2019-Feb 2022, http://www.akcchf.org/) and the State of North Carolina. Portions of this project were also completed while EL's research was supported by the Comparative Medicine and Translational Research Program of the National Institutes of Health under award number T32OD011130. The funders had no role in study design, data collection and analysis, decision to publish, or preparation of the manuscript.

Institutional Review Board Statement: Not applicable.

Informed Consent Statement: Not applicable.

Data Availability Statement: Data is available via Dryad (https://doi.org/10.5061/dryad.h18931zm1 accessed on 25 June 2020).

Acknowledgments: We thank Cristina Lanzas for helpful discussions relative to assessing sensitivity and specificity of the diagnostic assays used in this study and Sean Kent for assisting with the literature review of diagnostics used to assess bartonellosis in dogs. We also thank Cécile Ané, Dörte Döpfer, and Jingcheng Xu for discussions on latent class analysis methods.

Conflicts of Interest: In conjunction with Sushama Sontakke and North Carolina State University, Edward B. Breitschwerdt, DVM holds U.S. Patent No. 7,115,385: "Media and Methods for Cultivation of Microorganisms", which was issued 3 October 2006. He is a co-founder, shareholder and Chief Scientific Officer for Galaxy Diagnostics, a company that provides advanced diagnostic testing for the detection of *Bartonella* species infections. Maggi is the Chief Technical Officer and also a shareholder and founder of Galaxy Diagnostics. All other authors declare no potential conflicts of interest.

Abbreviations

hemangiosarcoma (HSA), composite reference standard (CRS), latent class analysis (LCA), random effects latent class analysis (RE-LCA), immunofluorescence assay (IFA), western immunoblot (WB), droplet digital PCR (ddPCR), quantitative PCR (qPCR), cat scratch disease (CSD), Bartonella alpha-proteobacteria growth medium (BAPGM), formalin-fixed paraffin embedded (FFPE), Canine Comparative Oncology and Genomics Consortium (CCOGC), intragenic transcribed spacer (ITS), 95% confidence interval (95% CI)

References

1. Breitschwerdt, E.B.; Kordick, D.L.; Malarkey, D.E.; Keene, B.; Hadfield, T.L.; Wilson, K. Endocarditis in a Dog Due to Infection with a Novel Bartonella Subspecies. *J. Clin. Microbiol.* **1995**, *33*, 154–160. [CrossRef]
2. Daly, J.S.; Worthington, M.G.; Brenner, D.O.N.J.; Moss, C.W.; Hollis, D.G.; Weyant, R.S.; Steigerwalt, A.G.; Weaver, R.E.; Daneshvar, M.I.; Connor, S.P.O. Rochalimaea Elizabethae Sp. Nov. Isolated from a Patient with Endocarditis. *J. Clin. Microbiol.* **1993**, *31*, 872–881. [CrossRef]
3. Hadfield, T.L.; Warren, R.; Kass, M.; Brun, E.; Levy, C. Endocarditis Caused by Rochalimaea Henselae. *Hum. Pathol.* **1993**, *24*, 1140–1141. [CrossRef]
4. Rodino, K.G.; Stone, E.; Saleh, O.A.; Theel, E.S. The Brief Case: Bartonella Henselae Endocarditis—A Case of Delayed Diagnosis. *J. Clin. Microbiol.* **2019**, *57*, e00114-19. [PubMed]
5. Golly, E.; Breitschwerdt, E.B.; Balakrishnan, N.; Moore, D.; Bizikova, P. Bartonella Henselae, Bartonella Koehlerae, and Rickettsia Rickettsii Seroconversion and Seroreversion in a Dog with Acute-Onset Fever, Lameness, and Lymphadenopathy Followed by a Protracted Disease Course. *Vet. Parasitol. Reg. Stud. Rep.* **2016**, *7*, 19–24. [CrossRef] [PubMed]
6. Cheslock, M.A.; Embers, M.E. Human Bartonellosis: An Underappreciated Public Health Problem? *Trop. Med. Infect. Dis.* **2019**, *4*, 69. [CrossRef] [PubMed]
7. Álvarez-Fernández, A.; Breitschwerdt, E.B.; Solano-Gallego, L. Bartonella Infections in Cats and Dogs Including Zoonotic Aspects. *Parasites Vectors* **2018**, *11*, 1–21. [CrossRef]
8. Scott, M.A.; McCurley, T.L.; Vnencak-Jones, C.L.; Hager, C.; McCoy, J.A.; Anderson, B.; Collins, R.D.; Edwards, K.M. Cat Scratch Disease: Detection of Bartonella Henselae DNA in Archival Biopsies from Patients with Clinically, Serologically, and Histologically Defined Disease. *Am. J. Pathol.* **1996**, *149*, 2161–2167. [PubMed]
9. Regnery, R.L.; Olson, J.G.; Perkins, B.A.; Bibb, W. Serological Response to "Rochalimaea Henselae" Antigen in Suspected Cat-Scratch Disease. *Lancet* **1992**, *339*, 1443–1445. [CrossRef]
10. Anderson, B.; Sims, K.; Regnery, R.; Robinson, L.; Schmidt, M.J.; Goral, S.; Hager, C.; Edwards, K. Detection of Rochalimaea Henselae DNA in Specimens from Cat Scratch Disease Patients by PCR. *J. Clin. Microbiol.* **1994**, *32*, 942–948. [CrossRef]

11. Rosas, L.; Rao, K.; McGough, C.; Becker, A. A Rare Case of Bartonella Encephalitis With Hemiplegia. *Child Neurol. Open* **2019**, *6*, 2329048X19826480. [CrossRef] [PubMed]
12. Breitschwerdt, E.B.; Greenberg, R.; Maggi, R.; Mozayeni, B.R.; Lewis, A.; Bradley, J. Bartonella Henselae Blood Stream Infection in a Boy with Pediatric Acute-Onset Neuropsychiatric Syndrome (PANS). *J. Cent. Nerv. Syst. Dis.* **2019**, *11*, 1179573519832014. [CrossRef] [PubMed]
13. Lam, J.C.; Fonseca, K.; Pabbaraju, K.; Meatherall, B.L. Case Report: Bartonella Quintana Endocarditis Outside of the Europe–African Gradient: Comprehensive Review of Cases within North America. *Am. J. Trop. Med. Hyg.* **2019**, *100*, 1125–1129. [CrossRef] [PubMed]
14. Edouard, S.; Nabet, C.; Lepidi, H.; Fournier, P.-E.; Raoult, D. Bartonella, a Common Cause of Endocarditis: A Report on 106 Cases and Review. *J. Clin. Microbiol.* **2015**, *53*, 824–829. [CrossRef] [PubMed]
15. La Scola, B.; Raoult, D. Culture of Bartonella Quintana and Bartonella Henselae from Human Samples: A 5-Year Experience (1993 to 1998). *J. Clin. Microbiol.* **1999**, *37*, 1899–1905. [CrossRef]
16. Theonest, N.O.; Carter, R.W.; Amani, N.; Doherty, S.L.; Hugho, E.; Keyyu, J.D.; Mable, B.K.; Shirima, G.M.; Tarimo, R.; Thomas, K.M.; et al. Molecular Detection and Genetic Characterization of Bartonella Species from Rodents and Their Associated Ectoparasites from Northern Tanzania. *PLoS ONE* **2019**, *14*, e0223667. [CrossRef] [PubMed]
17. Diaz, M.H.; Bai, Y.; Malania, L.; Winchell, J.M.; Kosoy, M. Development of a Novel Genus-Specific Real-Time PCR Assay for Detection and Differentiation of Bartonella Species and Genotypes. *J. Clin. Microbiol.* **2012**, *50*, 1645–1649. [CrossRef] [PubMed]
18. Allizond, V.; Costa, C.; Sidoti, F.; Scutera, S.; Bianco, G.; Sparti, R.; Banche, G.; Dalmasso, P.; Cuffini, A.M.; Cavallo, R.; et al. Serological and Molecular Detection of Bartonella Henselae in Specimens from Patients with Suspected Cat Scratch Disease in Italy: A Comparative Study. *PLoS ONE* **2019**, *14*, e0211945. [CrossRef] [PubMed]
19. Vermeulen, M.J.; Verbakel, H.; Notermans, D.W.; Reimerink, J.H.J.; Peeters, M.F. Evaluation of Sensitivity, Specificity and Cross-Reactivity in Bartonella Henselae Serology. *J. Med. Microbiol.* **2010**, *59*, 743–745. [CrossRef]
20. Vermeulen, M.J.; Herremans, M.; Verbakel, H.; Bergmans, A.M.C.; Roord, J.J.; van Dijken, P.J.; Peeters, M.F. Serological Testing for Bartonella Henselae Infections in The Netherlands: Clinical Evaluation of Immunofluorescence Assay and ELISA. *Clin. Microbiol. Infect.* **2007**, *13*, 627–634. [CrossRef]
21. Bergmans, A.M.C.; Peeters, M.F.; Schellekens, J.F.P.; Vos, M.C.; Sabbe, L.J.M.; Ossewaarde, J.M.; Verbakel, H.; Hooft, H.J.; Schouls, L.M. Pitfalls and Fallacies of Cat Scratch Disease Serology: Evaluation of Bartonella Henselae-Based Indirect Fluorescence Assay and Enzyme-Linked Immunoassay. *J. Clin. Microbiol.* **1997**, *35*, 1931–1937. [CrossRef]
22. Dupon, M.; Savin De Larclause, A.M.; Brouqui, P.; Drancourt, M.; Raoult, D.; De Mascarel, A.; Lacut, J.Y. Evaluation of Serological Response to Bartonella Henselae, Bartonella Quintana and Afipia Felis Antigens in 64 Patients with Suspected Cat-Scratch Disease. *Scand. J. Infect. Dis.* **1996**, *28*, 361–366. [CrossRef] [PubMed]
23. Sander, A.; Posselt, M.; Oberle, K.; Bredt, W. Seroprevalence of Antibodies to Bartonella Henselae in Patients with Cat Scratch Disease and in Healthy Controls: Evaluation and Comparison of Two Commercial Serological Tests. *Clin. Diagn. Lab. Immunol.* **1998**, *5*, 486–490. [CrossRef]
24. Drummond, M.R.; dos Santos, L.S.; Silva, M.N.D.; Almeida, A.R.D.; Diniz, P.P.V.D.P.; Angerami, R.; Velho, P.E.N.F. False Negative Results in Bartonellosis Diagnosis. *Vector-Borne Zoonotic Dis.* **2019**, *19*, 453–454. [CrossRef]
25. Mazzitelli, M.; Lamberti, A.G.; Quirino, A.; Marascio, N.; Barreca, G.S.; Costa, C.; Pisani, V.; Strazzulla, A.; Greco, G.; Liberto, M.C.; et al. Utility of Molecular Identification and Quantitation of Bartonella Species with Species-Specific Real-Time PCR for Monitoring Treatment Response: A Case Series. *Open Microbiol. J.* **2018**, *12*, 148–153. [CrossRef]
26. Breitschwerdt, E.B.; Maggi, R.G. Bartonella Quintana and Bartonella Vinsonii Subsp. Vinsonii Bloodstream Co-Infection in a Girl from North Carolina, USA. *Med. Microbiol. Immunol.* **2019**, *208*, 101–107. [CrossRef]
27. Yanagihara, M.; Tsuneoka, H.; Tanimoto, A.; Otsuyama, K.I.; Nishikawa, J.; Matsui, T.; Nojima, J.; Ichihara, K. Bartonella Henselae DNA in Seronegative Patients with Cat-Scratch Disease. *Emerg. Infect. Dis.* **2018**, *24*, 924–925. [CrossRef] [PubMed]
28. Breitschwerdt, E.B.; Bradley, J.M.; Maggi, R.G.; Lashnits, E.; Reicherter, P. Bartonella Associated Cutaneous Lesions (BACL) in People with Neuropsychiatric Symptoms. *Pathogens* **2020**, *9*, 1023. [CrossRef] [PubMed]
29. Deng, H.; Pang, Q.; Zhao, B.; Vayssier-Taussat, M. Molecular Mechanisms of Bartonella and Mammalian Erythrocyte Interactions: A Review. *Front. Cell. Infect. Microbiol.* **2018**, *8*, 431. [CrossRef] [PubMed]
30. Fung, H.L.; Calzada, J.; Saldaña, A.; Santamaria, A.M.; Pineda, V.; Gonzalez, K.; Chaves, L.F.; Garner, B.; Gottdenker, N. Domestic Dog Health Worsens with Socio-Economic Deprivation of Their Home Communities. *Acta Trop.* **2014**, *135*, 67–74. [CrossRef]
31. Calzada, J.E.; Saldaña, A.; González, K.; Rigg, C.; Pineda, V.; Santamaría, A.M.; Rodríguez, I.; Gottdenker, N.L.; Laurenti, M.D.; Chaves, L.F. Cutaneous Leishmaniasis in Dogs: Is High Seroprevalence Indicative of a Reservoir Role? *Parasitology* **2015**, *142*, 1202–1214. [CrossRef]
32. Cosford, K.L. Brucella Canis: An Update on Research and Clinical Management. *Can. Vet. J.* **2018**, *59*, 74–81. [PubMed]
33. Gunstra, A.; Steurer, J.A.; Seibert, R.L.; Dixon, B.C.; Russell, D.S. Sensitivity of Serologic Testing for Dogs Diagnosed with Coccidioidomycosis on Histology: 52 Cases (2012–2013). *J. Am. Anim. Hosp. Assoc.* **2019**, *55*, 238–242. [CrossRef] [PubMed]
34. Rosypal, A.C.; Troy, G.C.; Duncan, R.B.; Zajac, A.M.; Lindsay, D.S. Utility of Diagnostic Tests Used in Diagnosis of Infection in Dogs Experimentally Inoculated with a North American Isolate of Leishmania Infantum Infantum. *J. Vet. Intern. Med.* **2005**, *19*, 802–809. [CrossRef] [PubMed]

35. Mendonça, I.L.D.; Batista, J.F.; Schallig, H.; Alonso, D.P.; Ribolla, P.E.M.; Costa, D.L.; Costa, C.H.N. The Performance of Serological Tests for Leishmania Infantum Infection Screening in Dogs Depends on the Prevalence of the Disease. *Rev. Inst. Med. Trop. Sao Paulo* **2017**, *59*, e39. [CrossRef] [PubMed]
36. Portillo, A.; Maggi, R.; Oteo, J.A.; Bradley, J.; García-Álvarez, L.; San-Martín, M.; Roura, X.; Breitschwerdt, E. Bartonella Spp. Prevalence (Serology, Culture, and PCR) in Sanitary Workers in La Rioja Spain. *Pathogens* **2020**, *9*, 189. [CrossRef]
37. Pitassi, L.H.U.; de Paiva Diniz, P.P.V.; Scorpio, D.G.; Drummond, M.R.; Lania, B.G.; Barjas-Castro, M.L.; Gilioli, R.; Colombo, S.; Sowy, S.; Breitschwerdt, E.B.; et al. Bartonella Spp. Bacteremia in Blood Donors from Campinas, Brazil. *PLoS Negl. Trop. Dis.* **2015**, *9*, e0003467. [CrossRef]
38. Maggi, R.G.; Mascarelli, P.E.; Pultorak, E.L.; Hegarty, B.C.; Bradley, J.M.; Mozayeni, B.R.; Breitschwerdt, E.B. Bartonella Spp. Bacteremia in High-Risk Immunocompetent Patients. *Diagn. Microbiol. Infect. Dis.* **2011**, *71*, 430–437. [CrossRef]
39. Lashnits, E.; Correa, M.; Hegarty, B.C.; Birkenheuer, A.; Breitschwerdt, E.B. Bartonella Seroepidemiology in Dogs from North America, 2008–2014. *J. Vet. Intern. Med.* **2018**, *32*, 222–223. [CrossRef]
40. Breitschwerdt, E. Bartonellosis: One Health Perspectives for an Emerging Infectious Disease. *ILAR J.* **2014**, *55*, 46–58. [CrossRef]
41. Bowser, N.; Anderson, N.; Bowser, N.H.; Anderson, N.E. Dogs (Canis Familiaris) as Sentinels for Human Infectious Disease and Application to Canadian Populations: A Systematic Review. *Vet. Sci.* **2018**, *5*, 83. [CrossRef]
42. Chomel, B.B.; Kasten, R.W.; Williams, C.; Wey, A.C.; Henn, J.B.; Maggi, R.; Carrasco, S.; Mazet, J.; Boulouis, H.J.; Maillard, R.; et al. Bartonella Endocarditis: A Pathology Shared by Animal Reservoirs and Patients. *Ann. N. Y. Acad. Sci.* **2009**, *1166*, 120–126. [CrossRef]
43. Breitschwerdt, E. Bartonellosis, one Health and All Creatures Great and Small. *Vet. Dermatol.* **2017**, *28*, 111–121. [CrossRef]
44. Neupane, P.; Hegarty, B.C.; Marr, H.S.; Maggi, R.G.; Birkenheuer, A.J.; Breitschwerdt, E.B. Evaluation of Cell Culture-Grown *Bartonella* Antigens in Immunofluorescent Antibody Assays for the Serological Diagnosis of Bartonellosis in Dogs. *J. Vet. Intern. Med.* **2018**, *32*, 1958–1964. [CrossRef] [PubMed]
45. Duncan, A.W.; Maggi, R.G.; Breitschwerdt, E.B. A Combined Approach for the Enhanced Detection and Isolation of Bartonella Species in Dog Blood Samples: Pre-Enrichment Liquid Culture Followed by PCR and Subculture onto Agar Plates. *J. Microbiol. Methods* **2007**, *69*, 273–281. [CrossRef]
46. Perez, C.; Maggi, R.G.; Diniz, P.P.V.P.; Breitschwerdt, E.B. Molecular and Serological Diagnosis of Bartonella Infection in 61 Dogs from the United States. *J. Vet. Intern. Med.* **2011**, *25*, 805–810. [CrossRef]
47. Lashnits, E.; Neupane, P.; Bradley, J.M.; Richardson, T.; Thomas, R.; Linder, K.E.; Breen, M.; Maggi, R.G.; Breitschwerdt, E.B. Molecular Prevalence of Bartonella, Babesia, and Hemotropic Mycoplasma Species in Dogs with Hemangiosarcoma from across the United States. *PLoS ONE* **2020**, *15*, e0227234. [CrossRef]
48. Santilli, R.A.; Grego, E.; Battaia, S.; Gianella, P.; Tursi, M.; Di Girolamo, N.; Biasato, I.; Perego, M. Prevalence of Selected Cardiotropic Pathogens in the Myocardium of Adult Dogs with Unexplained Myocardial and Rhythm Disorders or with Congenital Heart Disease. *J. Am. Vet. Med. Assoc.* **2019**, *255*, 1150–1160. [CrossRef] [PubMed]
49. Lashnits, E.; Neupane, P.; Maggi, R.G.; Linder, K.E.; Bradley, J.M.; Balakrishnan, N.; Southern, B.L.; McKeon, G.P.; Chandrashekar, R.; Breitschwerdt, E.B. Detection of Bartonella Spp. in Dogs after Infection with Rickettsia Rickettsii. *J. Vet. Intern. Med.* **2020**, *34*, 145–159. [CrossRef]
50. Hegarty, B.C.; Bradley, J.M.; Lappin, M.R.; Balakrishnan, N.; Mascarelli, P.E.; Breitschwerdt, E.B. Analysis of Seroreactivity against Cell Culture-Derived Bartonella Spp. Antigens in Dogs. *J. Vet. Intern. Med.* **2014**, *28*, 38–41. [CrossRef] [PubMed]
51. Balakrishnan, N.; Cherry, N.; Linder, K.E.; Pierce, E.; Sontakke, N.; Hegarty, B.C.; Bradley, J.M.; Maggi, R.G.; Breitschwerdt, E.B. Experimental Infection of Dogs with Bartonella Henselae and Bartonella Vinsonii Subsp. Berkhoffii. *Vet. Immunol. Immunopathol.* **2013**, *156*, 153–158. [CrossRef]
52. Jensen, W.A.; Fall, M.Z.; Rooney, J.; Kordick, D.L.; Breitschwerdt, E.B. Rapid Identification and Differentiation of Bartonella Species Using a Single-Step PCR Assay. *J. Clin. Microbiol.* **2000**, *38*, 1717–1722. [CrossRef]
53. Breitschwerdt, E.B.; Maggi, R.G.; Duncan, A.W.; Nicholson, W.L.; Hegarty, B.C.; Woods, C.W. Bartonella Species in Blood of Immunocompetent Persons with Animal and Arthropod Contact. *Emerg. Infect. Dis.* **2007**, *13*, 938–941. [CrossRef]
54. Maggi, R.G.; Mascarelli, P.E.; Havenga, L.N.; Naidoo, V.; Breitschwerdt, E.B. Co-Infection with Anaplasma Platys, Bartonella Henselae and Candidatus Mycoplasma Haematoparvum in a Veterinarian. *Parasites Vectors* **2013**, *6*, 103. [CrossRef]
55. Maggi, R.G.; Breitschwerdt, E.B. Potential Limitations of the 16S-23S RRNA Intergenic Region for Molecular Detection of Bartonella Species. *J. Clin. Microbiol.* **2005**, *43*, 1171–1176. [CrossRef]
56. Neupane, P.; Sevala, S.; Balakrishnan, N.; Marr, H.; Wilson, J.; Maggi, R.; Birkenheuer, A.; Lappin, M.; Chomel, B.; Breitschwerdt, E.B. Validation of *Bartonella Henselae* Western Immunoblotting for Serodiagnosis of Bartonelloses in Dogs. *J. Clin. Microbiol.* **2020**, *58*, e01335-19. [CrossRef]
57. Breitschwerdt, E.B.; Suksawat, J.; Chomel, B.; Hegarty, B.C. The Immunologic Response of Dogs to Bartonella Vinsonii Subspecies Berkhoffii Antigens: As Assessed by Western Immunoblot Analysis. *J. Vet. Diagn. Investig.* **2003**, *15*, 349–354. [CrossRef] [PubMed]
58. Maggi, R.G.; Richardson, T.; Breitschwerdt, E.B.; Miller, J.C. Development and Validation of a Droplet Digital PCR Assay for the Detection and Quanti Fi Cation of Bartonella Species within Human Clinical Samples. *J. Microbiol. Methods* **2020**, *176*, 106022. [CrossRef] [PubMed]

59. Collins, J.; Albert, P.S. Estimating Diagnostic Accuracy without a Gold Standard: A Continued Controversy. *J. Biopharm. Stat.* **2016**, *26*, 1078–1082. [CrossRef] [PubMed]
60. Hui, S.L.; Zhou, X.H. Evaluation of Diagnostic Tests without Gold Standards. *Stat. Methods Med. Res.* **1998**, *7*, 354–370. [CrossRef] [PubMed]
61. Johnson, W.O.; Gastwirth, J.L.; Pearson, L.M. Screening without a 'Gold Standard'; The Hui-Walter Paradigm Revisited. *Am. J. Epidemiol.* **2001**, *153*, 921–924. [CrossRef]
62. Chamberlin, J.; Laughlin, L.; Gordon, S.; Romero, S.; Solórzano, N.; Regnery, R.L. Serodiagnosis of Bartonella Bacilliformis Infection by Indirect Fluorescence Antibody Assay: Test Development and Application to a Population in an Area of Bartonellosis Endemicity. *J. Clin. Microbiol.* **2000**, *38*, 4269–4271. [CrossRef] [PubMed]
63. Angkasekwinai, N.; Atkins, E.H.; Romero, S.; Grieco, J.; Chao, C.C.; Ching, W.M. An Evaluation Study of Enzyme-Linked Immunosorbent Assay (ELISA) Using Recombinant Protein Pap31 for Detection of Antibody against Bartonella Bacilliformis Infection among the Peruvian Population. *Am. J. Trop. Med. Hyg.* **2014**, *90*, 690–696. [CrossRef]
64. Wilson, I.G. MINIREVIEW Inhibition and Facilitation of Nucleic Acid Amplification. *Appl Env Micro.* **1997**, *63*, 3741–3751. [CrossRef] [PubMed]
65. Southern, B.L.; Neupane, P.; Ericson, M.E.; Dencklau, J.C.; Linder, K.E.; Bradley, J.M.; McKeon, G.P.; Long, C.T.; Breitschwerdt, E.B. *Bartonella Henselae* in a Dog with Ear Tip Vasculitis. *Vet. Dermatol.* **2018**, *29*, 537-e180. [CrossRef]
66. Pultorak, E.L.; Maggi, R.G.; Mascarelli, P.E.; Breitschwerdt, E.B. Serial Testing from a 3-Day Collection Period by Use of the Bartonella Alphaproteobacteria Growth Medium Platform May Enhance the Sensitivity of Bartonella Species Detection in Bacteremic Human Patients. *J. Clin. Microbiol.* **2013**, *51*, 1673–1677. [CrossRef]
67. Balakrishnan, N.; Musulin, S.; Varanat, M.; Bradley, J.M.; Breitschwerdt, E.B. Serological and Molecular Prevalence of Selected Canine Vector Borne Pathogens in Blood Donor Candidates, Clinically Healthy Volunteers, and Stray Dogs in North Carolina. *Parasites Vectors* **2014**, *7*, 116. [CrossRef] [PubMed]
68. Maes, R.K.; Langohr, I.M.; Wise, A.G.; Smedley, R.C.; Thaiwong, T.; Kiupel, M. Beyond H&E: Integration of Nucleic Acid-Based Analyses Into Diagnostic Pathology. *Vet. Pathol.* **2014**, *51*, 238–256.
69. Cao, Z.; Wu, W.; Wei, H.; Gao, C.; Zhang, L.; Wu, C.; Hou, L. Using Droplet Digital PCR in the Detection of Mycobacterium Tuberculosis DNA in FFPE Samples. *Int. J. Infect. Dis.* **2020**, *99*, 77–83. [CrossRef]
70. Tsukamoto, K.; Shinzawa, N.; Kawai, A.; Suzuki, M.; Kidoya, H.; Takakura, N.; Yamaguchi, H.; Kameyama, T.; Inagaki, H.; Kurahashi, H.; et al. The Bartonella Autotransporter BafA Activates the Host VEGF Pathway to Drive Angiogenesis. *Nat. Commun.* **2020**, *11*, 1–14. [CrossRef]
71. Eicher, S.C.; Dehio, C. Bartonella Entry Mechanisms into Mammalian Host Cells. *Cell. Microbiol.* **2012**, *14*, 1166–1173. [CrossRef] [PubMed]
72. Pérez Vera, C.; Diniz, P.P.V.P.P.; Pultorak, E.L.; Maggi, R.G.; Breitschwerdt, E.B. An Unmatched Case Controlled Study of Clinicopathologic Abnormalities in Dogs with Bartonella Infection. *Comp. Immunol. Microbiol. Infect. Dis.* **2013**, *36*, 481–487. [CrossRef]
73. Sykes, J.E.; Westropp, J.L.; Kasten, R.W.; Chomel, B.B. Association between Bartonella Species Infection and Disease in Pet Cats as Determined Using Serology and Culture. *J. Feline Med. Surg.* **2010**, *12*, 631–636. [CrossRef] [PubMed]
74. Brenner, E.C.; Chomel, B.B.; Singhasivanon, O.-U.; Namekata, D.Y.; Kasten, R.W.; Kass, P.H.; Cortés-Vecino, J.; Gennari, S.M.; Rajapakse, R.P.; Huong, L.T.; et al. Bartonella Infection in Urban and Rural Dogs from the Tropics: Brazil, Colombia, Sri Lanka and Vietnam. *Epidemiol. Infect.* **2012**, *141*, 1–8.
75. Cherry, N.; Diniz, P.; Maggi, R.; Hummel, J.; Hardie, E.; Behrend, E.; Rozanski, E.; DeFrancesco, T.; Cadenas, M.; Breitschwerdt, E. Isolation or Molecular Detection of Bartonella Henselae and Bartonella Vinsonii Subsp. Berkhoffii from Dogs with Idiopathic Cavitary Effusions. *J. Vet. Intern. Med.* **2009**, *23*, 186–189. [CrossRef] [PubMed]
76. Flor, M.; Weiß, M.; Selhorst, T.; Müller-Graf, C.; Greiner, M. Comparison of Bayesian and Frequentist Methods for Prevalence Estimation under Misclassification. *BMC Public Health* **2020**, *20*, 1–10. [CrossRef] [PubMed]
77. McV Messam, L.L.; Branscum, A.J.; Collins, M.T.; Gardner, I.A. Frequentist and Bayesian Approaches to Prevalence Estimation Using Examples from Johne's Disease. *Anim. Health Res. Rev. Conf. Res. Work. Anim. Dis.* **2008**, *9*, 1–23. [CrossRef]
78. Kostoulas, P.; Nielsen, S.S.; Branscum, A.J.; Johnson, W.O.; Dendukuri, N.; Dhand, N.K.; Toft, N.; Gardner, I.A. STARD-BLCM: Standards for the Reporting of Diagnostic Accuracy Studies That Use Bayesian Latent Class Models. *Prev. Vet. Med.* **2017**, *138*, 37–47. [CrossRef]
79. Josephson, K.L.; Gerba, C.P.; Pepper, I.L. Polymerase Chain Reaction Detection of Nonviable Bacterial Pathogens. *Appl. Environ. Microbiol.* **1993**, *59*, 3513–3515. [CrossRef]
80. Gutiérrez, R.; Vayssier-Taussat, M.; Buffet, J.-P.; Harrus, S. Guidelines for the Isolation, Molecular Detection, and Characterization of *Bartonella* Species. *Vector-Borne Zoonotic Dis.* **2017**, *17*, 42–50. [CrossRef] [PubMed]
81. Mazcko, C.; Thomas, R. The Establishment of the Pfizer-Canine Comparative Oncology and Genomics Consortium Biospecimen Repository. *Vet. Sci.* **2015**, *2*, 127–130. [CrossRef]
82. Breitschwerdt, E.B.; Maggi, R.G.; Quach, C.; Bradley, J.M. Bartonella Spp. Bloodstream Infection in a Canadian Family. *Vector-Borne Zoonotic Dis.* **2019**, *19*, 234–241. [CrossRef] [PubMed]
83. Varanat, M.; Maggi, R.G.; Linder, K.E.; Horton, S.; Breitschwerdt, E.B. Cross-Contamination in the Molecular Detection of *Bartonella* from Paraffin-Embedded Tissues. *Vet. Pathol.* **2009**, *46*, 940–944. [CrossRef] [PubMed]

84. Beath, K.J. Randomlca: An r Package for Latent Class with Random Effects Analysis. *J. Stat. Softw.* **2017**, *81*, 1–25. [CrossRef]
85. Sehgal, I.S.; Dhooria, S.; Aggarwal, A.N.; Behera, D.; Agarwal, R. Diagnostic Performance of Xpert MTB/RIF in Tuberculous Pleural Effusion: Systematic Review and Meta-Analysis. *J. Clin. Microbiol.* **2016**, *54*, 1133–1136. [CrossRef] [PubMed]
86. Buonfrate, D.; Marrone, R.; Silva, R.; Mirisola, C.; Ragusa, A.; Mistretta, M.; Perandin, F.; Bisoffi, Z. Prevalence of Strongyloidiasis in a Cohort of Migrants in Italy and Accuracy of a Novel Elisa Assay for s. Stercoralis Infection, a Cross-Sectional Study. *Microorganisms* **2021**, *9*, 401. [CrossRef]
87. McHugh, M.L. Interrater Reliability: The Kappa Statistic. *Biochem. Med.* **2012**, *22*, 276–282. [CrossRef]
